# Supplementary material for: Free energy perturbations in enzyme kinetic models reveal cryptic epistasis
Source: PLoS Comput Biol. 2026 Mar 11;22(3):e1013493. doi: 10.1371/journal.pcbi.1013493 (PMC12998945; doi:10.1371/journal.pcbi.1013493)
Supplement: S1 File — The Rmarkdown notebook outlining all of the code, with annotations, used to set up the simple kinetic model (outlined in the Methods and Fig 1). The document outlines the results and conclusions drawn from the simulation. (HTML) [file pcbi.1013493.s001.html]

Simple kinetic model simulations


Code 

- Show All Code
- Hide All Code

# Simple kinetic model simulations

# 1 Overview

We first set up a free energy diagram for the simple reaction E + S =
ES -> E + P. Then I extract rate and equilibrium constants using the
free energies, and make the assumption for rate constants that the
Arrhenius constant is *k*b \* T / *h*.

Next, we introduce 1000 random “mutations” which sample from -2 to 2
kcal mol-1 and modulate the energy of each ground and/or
transition state by that amount. These are all save in a spreadsheet
(see Supplementary data 1).

Finally, we test all combinations of the 1000 mutations and calculate
the predicted and observed rate constants.

# 2 Setup

First we need to setup the free energy profile based on the simple
reaction E + S = ES -> E + P

The free energies will be set up in the energies vector

```
energies <- list(e = 0,
                 e_s_dagger = 10,
                 es = -5,
                 es_dagger = 11
)
```

Then we define the global constants

```
kb = 3.297623483e-27 # kcal/K
h = 1.58e-37 # kcal s
temp = 273+25 # K in room temp
r = 1.9872036e-3 # kcal K-1 mol-1
A = (kb*temp)/h # Collision constant as defined broadly by transition state theory
```

We note that a more accurate approximation of the *A* constant
would utilize collision theory, due to the nature of E + S representing
a bimolecular collision of E + S. This means that the rate constant can
theoretically approach 6.2195683^{12} which exceeds rate-limiting
diffusion. Nevertheless, we retain this approximation for
simplicity.

Then we can define the equations for rate constants using the energy
terms:

```
define_constants <- function(energies) {
  kon = A*exp(-1*(energies$e_s_dagger-energies$e) / (r*temp)) # M s-1
  koff = A*exp(-1*(energies$e_s_dagger-energies$es) / (r*temp)) # s-1
  kchem = A*exp(-1*(energies$es_dagger-energies$es) / (r*temp)) # s-1
  
  kd = koff/kon
  kcat = kchem
  km = (koff+kchem)/kon
  catef = kcat/km
  
  return(list(kon = kon, 
              koff = koff, 
              kchem = kchem, 
              kd = kd, 
              kcat = kcat, 
              km = km,
              catef = catef))
}
```

And check the values for each constants (see **Table 1**
in the main text)

```
cur_const <- define_constants(energies)
cur_const
```

```
## $kon
## [1] 288414.7
## 
## $koff
## [1] 62.10776
## 
## $kchem
## [1] 11.4755
## 
## $kd
## [1] 0.0002153419
## 
## $kcat
## [1] 11.4755
## 
## $km
## [1] 0.0002551301
## 
## $catef
## [1] 44979.01
```

These seem like decent starting conditions for the experiment with
*k*on ~ 2.8e5, *k*off ~ 60,
*k*cat ~ 11, *K*M ~ 255 µM,
*K*D ~ 215 µM, and a *k*cat /
*K*M of 4.5e4

# 3 Mutational simulations

First we prepare a table to store the free energies and rate
constants of the mutants:

```
dt <- tibble(e = numeric(),
             e_s_dagger = numeric(),
             es = numeric(),
             es_dagger = numeric(),
             kon = numeric(),
             koff = numeric(),
             kchem = numeric(),
             kd = numeric(),
             kcat = numeric(),
             km = numeric(),
             catef = numeric())

dt <- rbind(dt, c(unlist(energies), unlist(cur_const)))
names(dt) = c("e",
              "e_s_dagger",
              "es",
              "es_dagger",
              "kon",
              "koff",
              "kchem",
              "kd",
              "kcat",
              "km",
              "catef")
dt
```

```
##   e e_s_dagger es es_dagger      kon     koff   kchem           kd    kcat
## 1 0         10 -5        11 288414.7 62.10776 11.4755 0.0002153419 11.4755
##             km    catef
## 1 0.0002551301 44979.01
```

## 3.1 Mutational sampling

Then we can setup a simulation where each mutant free energy is the
sum of the wt free energy and a randomly chosen energy ranging from -2
to 2 kcal mol-1

```
mutation <- function(dt){
  # Assuming mutation can change all steps by a random amount
  new_energies <- list(e = energies$e + runif(1, -2, 2),
                   e_s_dagger = energies$e_s_dagger + runif(1, -2, 2),
                   es = energies$es + runif(1, -2, 2),
                   es_dagger = energies$es_dagger + runif(1, -2, 2))
  mut_const <- define_constants(new_energies)
  
  return(rbind(dt, c(unlist(new_energies), unlist(mut_const))))
}
```

And we simulate 1000 mutations and export the data into
**Supplementary data 1**

```
for(i in 1:1000){
  dt <- mutation(dt)
}

head(dt)
```

```
##            e e_s_dagger        es es_dagger         kon         koff      kchem
## 1  0.0000000  10.000000 -5.000000 11.000000   288414.66   62.1077597  11.475499
## 2  1.8235750  11.749142 -6.047118 10.020295   327042.77    0.5526376  10.240775
## 3 -0.4379521   9.364719 -5.190478 10.159731   402469.45  131.6294029  34.380458
## 4 -0.1973071  11.226383 -4.577116 10.451364    26056.72   15.9911849  59.191495
## 5  1.0653539   8.180348 -4.631312  9.806529 37653611.58 2500.4487171 160.479158
## 6  0.5309521   9.614360 -5.837986 11.561836  1355883.65   28.9338805   1.079389
##             kd       kcat           km      catef
## 1 2.153419e-04  11.475499 2.551301e-04   44979.01
## 2 1.689802e-06  10.240775 3.300306e-05  310297.73
## 3 3.270544e-04  34.380458 4.124782e-04   83350.98
## 4 6.137068e-04  59.191495 2.885347e-03   20514.51
## 5 6.640661e-05 160.479158 7.066860e-05 2270869.47
## 6 2.133950e-05   1.079389 2.213558e-05   48762.61
```

```
write_csv(dt, "Supplementary Data 1.csv")
```

# 4 General statistics

We interrogate the spread of *K*D,
*k*cat, *K*M, and
*k*cat / *K*M for the 1000 single
mutants (see **Fig. 1b** in the main text)

```
dt_all_spread <- dt %>%
  mutate(kcat_norm = log10(kcat / kcat[1]),
         kd_norm = log10(kd / kd[1]),
         km_norm = log10(km / km[1]),
         catef_norm = log10(catef / catef[1])) %>%
  pivot_longer(c(kcat_norm, kd_norm, km_norm, catef_norm)) %>%
  select(name, value)

# Change density plot fill colors by groups
dt_all_spread_plot <- dt_all_spread %>%
  ggplot(aes(x = value, fill = name)) +
  geom_density() +
  scale_fill_manual(values = c("#745fe8","#ca3a7d","#ea692f","#f3b33e")) +
  geom_vline(xintercept = log10(1), col = "black", lty = 2) +
  xlim(c(-5, 5)) +
  labs(x = "log10 parameter value", y = "Density") +
  theme_classic()
  #theme(axis.line = element_line(size = 0.2, color = "black"), axis.ticks = element_line(size = 0.2, color = "black"), text = element_text(size = 9), axis.text = element_text(size = 8, color = "black"), legend.position = "none")

dt_all_spread_plot
```

```
#ggsave("density_plot_1.svg", plot = dt_all_spread_plot, width = 180/2, height = 247/4, dpi = 300, units = "mm")
```

# 5 Simulation of mutational combinations

Next, we create 106 double mutants by combining mutational
effects from each single mutant found in **S1 data**. We
compute the predicted kinetic parameters and observed parameters as
follows:

For predicted kinetic parameters, we take the product of the wt
kinetic parameter, the fold-change of mutation 1, and the fold-change of
mutation 2. For the observed kinetic parameter, we first compute each
microscopic rate constant of the double mutant by calculating the sum of
free energy changes to each state in the reaction coordinate based on
the single mutation effects. Then, we use the newly computed rate
constants to obtained the *true* kinetic parameter of the double
mutant.

We quantify epistasis as the ratio of the predicted vs observed
kinetic parameter, and consider it significant if it exceeds
1.5-fold.

For each kinetic parameter, we also collect information on the fold
change of the single mutation effect to determine whether the double
mutant exhibits sign or magnitude epistasis.

```
# Check for file, you can just upload if this is the case
if(file.exists('Supplementary Data 2.csv')){
  epi_dt <- read_csv("Supplementary Data 2.csv")
} else {
  # Generate all pairwise combinations of mutations
mut_combinations <- combn(2:(nrow(dt) - 1), 2, simplify = FALSE)

# Initialize a progress bar - ONLY WORKS IN CONSOLE, NOT IN RMD
pb <- progress_bar$new(
  format = "  Processing [:bar] :percent in :elapsed, ETA: :eta",
  total = length(mut_combinations),
  clear = FALSE,
  width = 60
)

# Process combinations with progress bar (console only)
epi_dt <- map_dfr(mut_combinations, function(pair) {
  pb$tick()  # Update progress bar at each iteration
  i <- pair[1]
  j <- pair[2]
  
  wt_catef <- dt$catef[1]
  mut1_fold_catef <- dt$catef[i] / wt_catef
  mut2_fold_catef <- dt$catef[j] / wt_catef
  
  pred_catef <- wt_catef * mut1_fold_catef * mut2_fold_catef
  
  wt_km <- dt$km[1]
  mut1_fold_km <- dt$km[i] / wt_km
  mut2_fold_km <- dt$km[j] / wt_km
  
  pred_km <- wt_km * mut1_fold_km * mut2_fold_km
  
  wt_kd <- dt$kd[1]
  mut1_fold_kd <- dt$kd[i] / wt_kd
  mut2_fold_kd <- dt$kd[j] / wt_kd
  
  pred_kd <- wt_kd * mut1_fold_kd * mut2_fold_kd
  
  wt_kcat <- dt$kcat[1]
  mut1_fold_kcat <- dt$kcat[i] / wt_kcat
  mut2_fold_kcat <- dt$kcat[j] / wt_kcat
  
  pred_kcat <- wt_kcat * mut1_fold_kcat * mut2_fold_kcat
  
  mut_e <- dt$e[1] + (dt$e[i] - dt$e[1] + dt$e[j] - dt$e[1])
  mut_e_s_dagger <- dt$e_s_dagger[1] + (dt$e_s_dagger[i] - dt$e_s_dagger[1] + dt$e_s_dagger[j] - dt$e_s_dagger[1])
  mut_es <- dt$es[1] + (dt$es[i] - dt$es[1] + dt$es[j] - dt$es[1])
  mut_es_dagger <- dt$es_dagger[1] + (dt$es_dagger[i] - dt$es_dagger[1] + dt$es_dagger[j] - dt$es_dagger[1])
  mut_e_p <- dt$e_p[1] + (dt$e_p[i] - dt$e_p[1] + dt$e_p[j] - dt$e_p[1])
  
  mut_energies <- list(e = mut_e, e_s_dagger = mut_e_s_dagger, es = mut_es, es_dagger = mut_es_dagger, e_p = mut_e_p)
  cur_mut_const <- define_constants(mut_energies)
  
  tibble(
    e = mut_energies$e,
    e_s_dagger = mut_energies$e_s_dagger,
    es = mut_energies$es,
    es_dagger = mut_energies$es_dagger,
    kon = cur_mut_const$kon,
    koff = cur_mut_const$koff,
    kchem = cur_mut_const$kchem,
    kd = cur_mut_const$kd,
    kcat = cur_mut_const$kcat,
    km = cur_mut_const$km,
    catef = cur_mut_const$catef,
    pred_catef = pred_catef,
    pred_km = pred_km,
    pred_kd = pred_kd,
    pred_kcat = pred_kcat,
    mut1_fold = mut1_fold_catef,
    mut2_fold = mut2_fold_catef,
    mut1_fold_km = mut1_fold_km,
    mut2_fold_km = mut2_fold_km,
    mut1_fold_kcat = mut1_fold_kcat,
    mut2_fold_kcat = mut2_fold_kcat,
    mut1_fold_kd = mut1_fold_kd,
    mut2_fold_kd = mut2_fold_kd,
    mut1_koff = dt$koff[i],
    mut2_koff = dt$koff[j],
    mut1_kchem = dt$kchem[i],
    mut2_kchem = dt$kchem[j],
    mut1 = i,
    mut2 = j
  )
})

head(epi_dt)

write_csv(epi_dt, "Supplementary Data 2.csv")
}
```

# 6 Investigation of mutational combinations

## 6.1 Significant epistasis in *k*cat / *K*M

First I establish a function to calculate significant epistasis and
classify it as magnitude, sign, and reciprocal sign

```
sig_epi_classification <- function(sig_thresh, epi_dt) {
  this_df <- epi_dt %>%
  mutate(sign_change = log10(catef/dt$catef[1]),
         sign_mut1 = log10(mut1_fold),
         sign_mut2 = log10(mut2_fold),
         epi = log10(catef/pred_catef)) %>%
  mutate(sign = case_when( ((epi > log10(sig_thresh) | epi < log10(1/sig_thresh)) & sign_mut1 > 0 & sign_mut2 > 0 & sign_change < 0) ~ "reciprocal",
                           ((epi > log10(sig_thresh) | epi < log10(1/sig_thresh)) & sign_mut1 < 0 & sign_mut2 < 0 & sign_change > 0) ~ "reciprocal",
                           ((epi > log10(sig_thresh) | epi < log10(1/sig_thresh)) & sign_mut1 > 0 & sign_mut2 < 0 & (sign_change > sign_mut1 | sign_change < sign_mut2) ) ~ "sign",
                           ((epi > log10(sig_thresh) | epi < log10(1/sig_thresh)) & sign_mut1 < 0 & sign_mut2 > 0 & (sign_change < sign_mut1 | sign_change > sign_mut2) ) ~ "sign",
                           (epi > log10(sig_thresh) | epi < log10(1/sig_thresh)) ~ "magnitude",
                           TRUE ~ "no epistasis"))
  
  return(this_df)
}
```

Then, we check much significant epistasis is there in
*k*cat / *K*M with a 1.5-fold
threshold?

```
epi_dt_2_sig <- sig_epi_classification(1.5, epi_dt)
epi_dt_2_sig %>% count(sign) %>% mutate(n / sum(n) * 100)
```

```
## # A tibble: 4 × 3
##   sign              n `n/sum(n) * 100`
##   <chr>         <int>            <dbl>
## 1 magnitude    164711           33.0  
## 2 no epistasis 302146           60.6  
## 3 reciprocal     3648            0.732
## 4 sign          27996            5.62
```

What about a 2-fold threshold?

```
epi_dt_2_sig <- sig_epi_classification(2, epi_dt)
epi_dt_2_sig %>% count(sign) %>% mutate(n / sum(n) * 100)
```

```
## # A tibble: 4 × 3
##   sign              n `n/sum(n) * 100`
##   <chr>         <int>            <dbl>
## 1 magnitude    115368           23.1  
## 2 no epistasis 355644           71.3  
## 3 reciprocal     3475            0.697
## 4 sign          24014            4.82
```

A 5-fold threshold?

```
epi_dt_2_sig <- sig_epi_classification(5, epi_dt)
epi_dt_2_sig %>% count(sign) %>% mutate(n / sum(n) * 100)
```

```
## # A tibble: 4 × 3
##   sign              n `n/sum(n) * 100`
##   <chr>         <int>            <dbl>
## 1 magnitude     25789            5.17 
## 2 no epistasis 463054           92.9  
## 3 reciprocal     1944            0.390
## 4 sign           7714            1.55
```

A 10-fold threshold?

```
epi_dt_2_sig <- sig_epi_classification(10, epi_dt)
epi_dt_2_sig %>% count(sign) %>% mutate(n / sum(n) * 100)
```

```
## # A tibble: 4 × 3
##   sign              n `n/sum(n) * 100`
##   <chr>         <int>            <dbl>
## 1 magnitude      7255            1.46 
## 2 no epistasis 488128           97.9  
## 3 reciprocal      932            0.187
## 4 sign           2186            0.439
```

Note that sign epistasis does not disappear even at high significance
thresholds

## 6.2 Significant epistasis in *k*cat

```
epi_dt %>%
  mutate(sign_change = log10(kcat/dt$kcat[1]),
         epi = log10(kcat/pred_kcat)) %>%
  mutate(sign = case_when( (epi > log10(1.5) | epi < log10(1/1.5)) ~ "epistasis",
                           TRUE ~ "no epistasis")
         
  ) %>%
  count(sign) %>% mutate(n / sum(n) * 100)
```

```
## # A tibble: 1 × 3
##   sign              n `n/sum(n) * 100`
##   <chr>         <int>            <dbl>
## 1 no epistasis 498501              100
```

## 6.3 Significant epistasis in *K*M

```
sig_epi_classification_km <- function(sig_thresh, epi_dt) {
  this_df <- epi_dt %>%
  mutate(sign_change = log10(km/dt$km[1]),
         sign_mut1 = log10(mut1_fold_km),
         sign_mut2 = log10(mut2_fold_km),
         epi = log10(km/pred_km)) %>%
  mutate(sign = case_when( ((epi > log10(sig_thresh) | epi < log10(1/sig_thresh)) & sign_mut1 > 0 & sign_mut2 > 0 & sign_change < 0) ~ "reciprocal",
                           ((epi > log10(sig_thresh) | epi < log10(1/sig_thresh)) & sign_mut1 < 0 & sign_mut2 < 0 & sign_change > 0) ~ "reciprocal",
                           ((epi > log10(sig_thresh) | epi < log10(1/sig_thresh)) & sign_mut1 > 0 & sign_mut2 < 0 & (sign_change > sign_mut1 | sign_change < sign_mut2) ) ~ "sign",
                           ((epi > log10(sig_thresh) | epi < log10(1/sig_thresh)) & sign_mut1 < 0 & sign_mut2 > 0 & (sign_change < sign_mut1 | sign_change > sign_mut2) ) ~ "sign",
                           (epi > log10(sig_thresh) | epi < log10(1/sig_thresh)) ~ "magnitude",
                           TRUE ~ "no epistasis"))
  
  return(this_df)
}
```

```
epi_dt_2_sig <- sig_epi_classification_km(1.5, epi_dt)
epi_dt_2_sig %>% count(sign) %>% mutate(n / sum(n) * 100)
```

```
## # A tibble: 4 × 3
##   sign              n `n/sum(n) * 100`
##   <chr>         <int>            <dbl>
## 1 magnitude    171418           34.4  
## 2 no epistasis 302146           60.6  
## 3 reciprocal     2065            0.414
## 4 sign          22872            4.59
```

At a 2-fold threshold?

```
epi_dt_2_sig <- sig_epi_classification_km(2, epi_dt)
epi_dt_2_sig %>% count(sign) %>% mutate(n / sum(n) * 100)
```

```
## # A tibble: 4 × 3
##   sign              n `n/sum(n) * 100`
##   <chr>         <int>            <dbl>
## 1 magnitude    122005           24.5  
## 2 no epistasis 355644           71.3  
## 3 reciprocal     1926            0.386
## 4 sign          18926            3.80
```

At a 5-fold threshold?

```
epi_dt_2_sig <- sig_epi_classification_km(5, epi_dt)
epi_dt_2_sig %>% count(sign) %>% mutate(n / sum(n) * 100)
```

```
## # A tibble: 4 × 3
##   sign              n `n/sum(n) * 100`
##   <chr>         <int>            <dbl>
## 1 magnitude     29374            5.89 
## 2 no epistasis 463054           92.9  
## 3 reciprocal     1005            0.202
## 4 sign           5068            1.02
```

At a 10-fold threshold?

```
epi_dt_2_sig <- sig_epi_classification_km(10, epi_dt)
epi_dt_2_sig %>% count(sign) %>% mutate(n / sum(n) * 100)
```

```
## # A tibble: 4 × 3
##   sign              n `n/sum(n) * 100`
##   <chr>         <int>            <dbl>
## 1 magnitude      8597           1.72  
## 2 no epistasis 488128          97.9   
## 3 reciprocal      402           0.0806
## 4 sign           1374           0.276
```

## 6.4 Significant epistasis in *K*D

```
epi_dt %>%
  mutate(sign_change = log10(kd/dt$kd[1]),
         epi = log10(kd/pred_kd)) %>%
  mutate(sign = case_when( (epi > log10(1.5) | epi < log10(1/1.5)) ~ "epistasis",
                           TRUE ~ "no epistasis")
         
  ) %>%
  count(sign) %>% mutate(n / sum(n) * 100)
```

```
## # A tibble: 1 × 3
##   sign              n `n/sum(n) * 100`
##   <chr>         <int>            <dbl>
## 1 no epistasis 498501              100
```

## 6.5 Correlation plots of predicted vs observed effects

What is the plot of *k*cat / *K*M
of predicted vs observed employing a 1.5-fold threshold?

```
epi_dt_2_sig <- sig_epi_classification(1.5, epi_dt)

epi_dt_2_sig_catef_plot <- epi_dt_2_sig %>%
  mutate(sign = factor(sign, levels = c("no epistasis", "magnitude", "sign", "reciprocal"))) %>%
  ggplot(aes(x = log10(pred_catef), y = log10(catef), color = sign)) +
  geom_point(alpha = 0.4, size = 0.5) +
  geom_abline(slope = 1, intercept = 0, linewidth = 0.3) +
  geom_hline(yintercept = log10(dt[1,]$catef), lty = 2, linewidth = 0.3) +
  geom_vline(xintercept = log10(dt[1,]$catef), lty = 2, linewidth = 0.3) +
  scale_color_manual(values = c("grey","#E69F00","#1f78b4","#d73027")) +
  labs(x = "Log10(Predicted kcat/Km)", "Log10(Observed kcat/Km)") +
  theme_classic() +
  theme(axis.line = element_line(size = 0.3, color = "black"), axis.ticks = element_line(size = 0.2, color = "black"), text = element_text(size = 9), axis.text = element_text(size = 8, color = "black"))
```

```
## Warning: The `size` argument of `element_line()` is deprecated as of ggplot2 3.4.0.
## ℹ Please use the `linewidth` argument instead.
## This warning is displayed once every 8 hours.
## Call `lifecycle::last_lifecycle_warnings()` to see where this warning was
## generated.
```

```
epi_dt_2_sig_catef_plot
```

```
##ggsave("catef_muts.tiff", plot = epi_dt_2_sig_catef_plot, width = 180/2, height = 247/4, dpi = 600, units = "mm")
```

What is the plot of *K*M predicted vs observed?

```
epi_dt_2_sig_km <- sig_epi_classification_km(1.5, epi_dt)

epi_dt_2_sig_km_plot <- epi_dt_2_sig_km %>%
  mutate(sign = factor(sign, levels = c("no epistasis", "magnitude", "sign", "reciprocal"))) %>%
  ggplot(aes(x = log10(pred_km), y = log10(km), color = sign)) +
  geom_point(alpha = 0.4, size = 0.5) +
  geom_abline(slope = 1, intercept = 0, linewidth = 0.3) +
  geom_hline(yintercept = log10(dt[1,]$km), lty = 2, linewidth = 0.3) +
  geom_vline(xintercept = log10(dt[1,]$km), lty = 2, linewidth = 0.3) +
  scale_color_manual(values = c("grey","#E69F00","#1f78b4","#d73027")) +
  labs(x = "Log10(Predicted Km)", "Log10(Observed Km)") +
  theme_classic() +
  theme(axis.line = element_line(size = 0.3, color = "black"), axis.ticks = element_line(size = 0.2, color = "black"), text = element_text(size = 9), axis.text = element_text(size = 8, color = "black"))

epi_dt_2_sig_km_plot
```

```
##ggsave("km_muts.tiff", plot = epi_dt_2_sig_km_plot, width = 180/2, height = 247/4, dpi = 600, units = "mm")
```

What is the plot of *k*cat predicted vs
observed?

```
epi_dt_2_sig_kcat <- epi_dt %>%
  mutate(sign_change = log10(kcat/dt$kcat[1]),
         epi = log10(kcat/pred_kcat)) %>%
  mutate(sign = case_when( (epi > log10(1.5) | epi < log10(1/1.5)) ~ "epistasis",
                           TRUE ~ "no epistasis"))

# View(dt %>% mutate(across(everything(), ~ . - first(.))))

epi_dt_2_sig_kcat_plot <- epi_dt_2_sig_kcat %>%
  ggplot(aes(x = log10(pred_kcat), y = log10(kcat), color = sign)) +
  geom_point(alpha = 0.4) +
  geom_abline(slope = 1, intercept = 0, linewidth = 0.3) +
  geom_hline(yintercept = log10(dt[1,]$kcat), lty = 2, linewidth = 0.3) +
  geom_vline(xintercept = log10(dt[1,]$kcat), lty = 2, linewidth = 0.3) +
  scale_color_manual(values = c("grey")) +
  labs(x = "Log10(Predicted kcat)", "Log10(Observed kcat)") +
  theme_classic() +
  theme(axis.line = element_line(size = 0.3, color = "black"), axis.ticks = element_line(size = 0.2, color = "black"), text = element_text(size = 9), axis.text = element_text(size = 8, color = "black"))

epi_dt_2_sig_kcat_plot
```

```
#ggsave("kcat_muts.tiff", plot = epi_dt_2_sig_kcat_plot, width = 180/2, height = 247/4, dpi = 600, units = "mm")
```

What is the plot of *K*D predicted vs observed?

```
epi_dt_2_sig_kd <- epi_dt %>%
  mutate(sign_change = log10(kd/dt$kd[1]),
         epi = log10(kd/pred_kd)) %>%
  mutate(sign = case_when( (epi > log10(1.5) | epi < log10(1/1.5)) ~ "epistasis",
                           TRUE ~ "no epistasis"))

# View(dt %>% mutate(across(everything(), ~ . - first(.))))

epi_dt_2_sig_kd_plot <- epi_dt_2_sig_kd %>%
  ggplot(aes(x = log10(pred_kd), y = log10(kd), color = sign)) +
  geom_point(alpha = 0.4) +
  geom_abline(slope = 1, intercept = 0, linewidth = 0.3) +
  geom_hline(yintercept = log10(dt[1,]$kd), lty = 2, linewidth = 0.3) +
  geom_vline(xintercept = log10(dt[1,]$kd), lty = 2, linewidth = 0.3) +
  scale_color_manual(values = c("grey")) +
  labs(x = "Log10(Predicted Kd)", "Log10(Observed Kd)") +
  theme_classic() +
  theme(axis.line = element_line(size = 0.3, color = "black"), axis.ticks = element_line(size = 0.2, color = "black"), text = element_text(size = 9), axis.text = element_text(size = 8, color = "black"))

epi_dt_2_sig_kd_plot
```

```
##ggsave("kd_muts.tiff", plot = epi_dt_2_sig_kd_plot, width = 180/2, height = 247/4, dpi = 600, units = "mm")
```

## 6.6 Positive-negative spread

What is the spread of positive and negative mutations for
*k*cat / *K*M

```
epi_dt %>%
  mutate(epi = log10(catef/pred_catef)) %>%
  filter(epi >= log10(1.5) | epi <= log10(1/1.5)) %>%
  mutate(posneg = case_when(epi > 0 ~ "positive",
                           epi < 0  ~ "negative",
                           TRUE ~ "neutral")) %>%
  count(posneg) %>%
  mutate(freq = n / sum(n) * 100)
```

```
## # A tibble: 2 × 3
##   posneg        n  freq
##   <chr>     <int> <dbl>
## 1 negative  78675  40.1
## 2 positive 117680  59.9
```

Then *K*M

```
epi_dt %>%
  mutate(epi = log10(km/pred_km)) %>%
  filter(epi >= log10(1.5) | epi <= log10(1/1.5)) %>%
  mutate(posneg = case_when(epi > 0 ~ "positive",
                           epi < 0  ~ "negative",
                           TRUE ~ "neutral")) %>%
  count(posneg) %>%
  mutate(freq = n / sum(n) * 100)
```

```
## # A tibble: 2 × 3
##   posneg        n  freq
##   <chr>     <int> <dbl>
## 1 negative 117680  59.9
## 2 positive  78675  40.1
```

# 7 Calculation of epistasis in Km

## 7.1 Epistasis for wt rate constants

First we create the function to calculate epistasis with the wt terms
and create a parameter grid for the fold-changes in the relevant rate
constants.

```
epistasis_in_km_rates_vectorized <- function(a1, a2, b1, b2, koff = 62.10776, kcat = 11.4755) {
  
  epi_km <- log10( (a1*a2*koff + b1*b2*kcat) / ( (a1*koff + b1*kcat)*(a2*koff + b2*kcat) / (koff + kcat)) )
  
  return(tibble(
    a1 = a1,
    a2 = a2,
    b1 = b1,
    b2 = b2,
    del_a1_b1 = log10(a1 / b1),
    del_a2_b2 = log10(a2 / b2),
    epi_km = epi_km
  ))
}

# Generate parameter grid efficiently
param_grid <- expand_grid(
  a1 = 10^seq(-2, 2, by = 0.1),
  a2 = 10^seq(-2, 2, by = 0.1),
  b1 = 10^seq(-2, 2, by = 0.1),
  b2 = 10^seq(-2, 2, by = 0.1),
)
```

Then we perform the simulation and aggregate results together,
merging duplicate measurements as an average

```
# Calculate epistasis for all combinations at once
epistasis_results <- epistasis_in_km_rates_vectorized(param_grid$a1, param_grid$a2, param_grid$b1, param_grid$b2)

results <- param_grid %>%
  bind_cols(epistasis_results %>% select(del_a1_b1, del_a2_b2, epi_km))

# Aggregate data to remove duplicates for plotting by taking epi_km mean and most drastic epistasis change
results_aggregated <- results %>%
  group_by(del_a1_b1, del_a2_b2) %>%
  summarise(
    epi_km = mean(epi_km, na.rm = TRUE),
    .groups = "drop"
  )
```

And we can visualize the variation in epistasis

```
# Create heatmap with epistasis
heatmap_plot <- results_aggregated %>%
  ggplot(aes(x = del_a1_b1, y = del_a2_b2)) +
  geom_tile(aes(fill = epi_km)) +
  scale_fill_gradient2(
    name = "log10 Epistasis in Km",
    low = "red",
    mid = "white", 
    high = "blue",
    midpoint = 0
  ) +
  labs(
    x = "log10 (a1 / b1)",
    y = "log10 (a2 / b2)",
  ) +
  theme_classic() +
  theme(axis.line = element_line(size = 0.3, color = "black"), 
        axis.ticks = element_line(size = 0.2, color = "black"), 
        text = element_text(size = 9), 
        axis.text = element_text(size = 8, color = "black"), 
        legend.position = "top")

# Display the plot
heatmap_plot
```

## 7.2 Plotting simulated data on the epistasis heatmap

```
epi_dt_inspection <- tibble(
    a1 = epi_dt_2_sig_km$mut1_koff/dt$koff[1],
    a2 = epi_dt_2_sig_km$mut2_koff/dt$koff[1],
    b1 = epi_dt_2_sig_km$mut1_kchem/dt$kchem[1],
    b2 = epi_dt_2_sig_km$mut2_kchem/dt$kchem[1],
    epi_km = log10(epi_dt_2_sig_km$km/epi_dt_2_sig_km$pred_km),
    sign = epi_dt_2_sig_km$sign
  )

epi_dt_inspection <- epi_dt_inspection %>%
  mutate(del_a1_b1 = log10(a1 / b1),
         del_a2_b2 = log10(a2 / b2)) %>%
  select(-c(a1, a2, b1, b2)) %>%
  mutate(sign = factor(sign, levels = c("no epistasis", "magnitude", "sign", "reciprocal")))

# Create heatmap with epistasis
heatmap_plot_2 <- results_aggregated %>%
  ggplot(aes(x = del_a1_b1, y = del_a2_b2)) +
  geom_tile(aes(fill = epi_km)) +
  geom_point(data = epi_dt_inspection, aes(x = del_a1_b1, y = del_a2_b2, color = sign),
             size = 0.05, alpha = 0.4) +
  scale_fill_gradient2(
    name = "log10 Epistasis in Km",
    low = "red",
    mid = "white", 
    high = "blue",
    midpoint = 0
  ) +
  scale_color_manual(values = c("grey","#fdae61","#1f78b4","#d73027")) +
  labs(
    x = "log10 (a1 / b1)",
    y = "log10 (a2 / b2)",
  ) +
  theme_classic() +
  theme(axis.line = element_line(size = 0.3, color = "black"), 
        axis.ticks = element_line(size = 0.2, color = "black"), 
        text = element_text(size = 9), 
        axis.text = element_text(size = 8, color = "black"), 
        legend.position = "top")

# Display the plot
heatmap_plot_2
```

## 7.3 Varying *k*-1 relative to *k*2

```
epistasis_in_km_rates_vectorized_varied <- function(a1, a2, b1, b2, koff, kcat) {
  
  epi_km <- log10( (a1*a2*koff + b1*b2*kcat) / ( (a1*koff + b1*kcat)*(a2*koff + b2*kcat) / (koff + kcat)) )
  
  return(tibble(
    a1 = a1,
    a2 = a2,
    b1 = b1,
    b2 = b2,
    del_a1_b1 = log10(a1 / b1),
    del_a2_b2 = log10(a2 / b2),
    epi_km = epi_km
  ))
}

# Calculate epistasis for all combinations at once
epistasis_results_1 <- epistasis_in_km_rates_vectorized_varied(param_grid$a1, param_grid$a2, param_grid$b1, param_grid$b2, 0.01*11.4755, 11.4755)
epistasis_results_2 <- epistasis_in_km_rates_vectorized_varied(param_grid$a1, param_grid$a2, param_grid$b1, param_grid$b2, 0.1*11.4755, 11.4755)
epistasis_results_3 <- epistasis_in_km_rates_vectorized_varied(param_grid$a1, param_grid$a2, param_grid$b1, param_grid$b2, 11.4755, 11.4755)
epistasis_results_4 <- epistasis_in_km_rates_vectorized_varied(param_grid$a1, param_grid$a2, param_grid$b1, param_grid$b2, 10*11.4755, 11.4755)
epistasis_results_5 <- epistasis_in_km_rates_vectorized_varied(param_grid$a1, param_grid$a2, param_grid$b1, param_grid$b2, 100*11.4755, 11.4755)

results_1 <- param_grid %>%
  bind_cols(epistasis_results_1 %>% select(del_a1_b1, del_a2_b2, epi_km)) %>%
  group_by(del_a1_b1, del_a2_b2) %>%
  summarise(
    epi_km = mean(epi_km, na.rm = TRUE),
    .groups = "drop"
  )

results_2 <- param_grid %>%
  bind_cols(epistasis_results_2 %>% select(del_a1_b1, del_a2_b2, epi_km)) %>%
  group_by(del_a1_b1, del_a2_b2) %>%
  summarise(
    epi_km = mean(epi_km, na.rm = TRUE),
    .groups = "drop"
  )

results_3 <- param_grid %>%
  bind_cols(epistasis_results_3 %>% select(del_a1_b1, del_a2_b2, epi_km)) %>%
  group_by(del_a1_b1, del_a2_b2) %>%
  summarise(
    epi_km = mean(epi_km, na.rm = TRUE),
    .groups = "drop"
  )

results_4 <- param_grid %>%
  bind_cols(epistasis_results_4 %>% select(del_a1_b1, del_a2_b2, epi_km)) %>%
  group_by(del_a1_b1, del_a2_b2) %>%
  summarise(
    epi_km = mean(epi_km, na.rm = TRUE),
    .groups = "drop"
  )

results_5 <- param_grid %>%
  bind_cols(epistasis_results_5 %>% select(del_a1_b1, del_a2_b2, epi_km)) %>%
  group_by(del_a1_b1, del_a2_b2) %>%
  summarise(
    epi_km = mean(epi_km, na.rm = TRUE),
    .groups = "drop"
  )
```

### 7.3.1 *k*-1 = 0.01(*k*2)

```
# Create heatmap with epistasis
heatmap_plot_0.01 <- results_1 %>%
  ggplot(aes(x = del_a1_b1, y = del_a2_b2)) +
  geom_tile(aes(fill = epi_km)) +
  scale_fill_gradient2(
    name = "log10 Epistasis in Km",
    low = "red",
    mid = "white", 
    high = "blue",
    midpoint = 0
  ) +
  labs(
    x = "log10 (a1 / b1)",
    y = "log10 (a2 / b2)",
  ) +
  theme_classic() +
  theme(axis.line = element_line(size = 0.3, color = "black"), 
        axis.ticks = element_line(size = 0.2, color = "black"), 
        text = element_text(size = 9), 
        axis.text = element_text(size = 8, color = "black"), 
        legend.position = "top")

# Display the plot
heatmap_plot_0.01
```

### 7.3.2 *k*-1 = 0.1(*k*2)

```
# Create heatmap with epistasis
heatmap_plot_0.1 <- results_2 %>%
  ggplot(aes(x = del_a1_b1, y = del_a2_b2)) +
  geom_tile(aes(fill = epi_km)) +
  scale_fill_gradient2(
    name = "log10 Epistasis in Km",
    low = "red",
    mid = "white", 
    high = "blue",
    midpoint = 0
  ) +
  labs(
    x = "log10 (a1 / b1)",
    y = "log10 (a2 / b2)",
  ) +
  theme_classic() +
  theme(axis.line = element_line(size = 0.3, color = "black"), 
        axis.ticks = element_line(size = 0.2, color = "black"), 
        text = element_text(size = 9), 
        axis.text = element_text(size = 8, color = "black"), 
        legend.position = "top")

# Display the plot
heatmap_plot_0.1
```

### 7.3.3 *k*-1 = *k*2

```
# Create heatmap with epistasis
heatmap_plot_equal <- results_3 %>%
  ggplot(aes(x = del_a1_b1, y = del_a2_b2)) +
  geom_tile(aes(fill = epi_km)) +
  scale_fill_gradient2(
    name = "log10 Epistasis in Km",
    low = "red",
    mid = "white", 
    high = "blue",
    midpoint = 0
  ) +
  labs(
    x = "log10 (a1 / b1)",
    y = "log10 (a2 / b2)",
  ) +
  theme_classic() +
  theme(axis.line = element_line(size = 0.3, color = "black"), 
        axis.ticks = element_line(size = 0.2, color = "black"), 
        text = element_text(size = 9), 
        axis.text = element_text(size = 8, color = "black"), 
        legend.position = "top")

# Display the plot
heatmap_plot_equal
```

### 7.3.4 *k*-1 = 10(*k*2)

```
# Create heatmap with epistasis
heatmap_plot_10 <- results_4 %>%
  ggplot(aes(x = del_a1_b1, y = del_a2_b2)) +
  geom_tile(aes(fill = epi_km)) +
  scale_fill_gradient2(
    name = "log10 Epistasis in Km",
    low = "red",
    mid = "white", 
    high = "blue",
    midpoint = 0
  ) +
  labs(
    x = "log10 (a1 / b1)",
    y = "log10 (a2 / b2)",
  ) +
  theme_classic() +
  theme(axis.line = element_line(size = 0.3, color = "black"), 
        axis.ticks = element_line(size = 0.2, color = "black"), 
        text = element_text(size = 9), 
        axis.text = element_text(size = 8, color = "black"), 
        legend.position = "top")

# Display the plot
heatmap_plot_10
```

### 7.3.5 *k*-1 = 100(*k*2)

```
# Create heatmap with epistasis
heatmap_plot_100 <- results_5 %>%
  ggplot(aes(x = del_a1_b1, y = del_a2_b2)) +
  geom_tile(aes(fill = epi_km)) +
  scale_fill_gradient2(
    name = "log10 Epistasis in Km",
    low = "red",
    mid = "white", 
    high = "blue",
    midpoint = 0
  ) +
  labs(
    x = "log10 (a1 / b1)",
    y = "log10 (a2 / b2)",
  ) +
  theme_classic() +
  theme(axis.line = element_line(size = 0.3, color = "black"), 
        axis.ticks = element_line(size = 0.2, color = "black"), 
        text = element_text(size = 9), 
        axis.text = element_text(size = 8, color = "black"), 
        legend.position = "top")

# Display the plot
heatmap_plot_100
```
