# Supplementary material for: Free energy perturbations in enzyme kinetic models reveal cryptic epistasis
Source: PLoS Comput Biol. 2026 Mar 11;22(3):e1013493. doi: 10.1371/journal.pcbi.1013493 (PMC12998945; doi:10.1371/journal.pcbi.1013493)
Supplement: S2 File — 1) Demonstration of conditions that create non-specific epistasis in the simple kinetic ensemble, 2) Justification for the lack of epistasis in kcat for the simple kinetic model, 3) Justification for the lack of epistasis in KD for all models. (DOCX) [file pcbi.1013493.s002.docx]

**Supplementary derivations**

**1. Demonstration of conditions that create non-specific epistasis in the kinetic ensemble**

***1.1 Definition of null-model and observed epistasis calculations in K_M_***

In the simple kinetic model, we define *K*_M_ as:

|  | $K_{M}=\frac{k_{-1}+k_{2}}{k_{1}}$ | (1) |
| --- | --- | --- |

And *k*_cat_ as:

|  | $k_{cat}=k_{2}$ | (2) |
| --- | --- | --- |

The effect of a mutation can perturb any of these three microscopic constants. Thus, we define the mutant scaling fold-changes as:

**S1 Table – Mutant 1 and 2 microscopic rate constants expressed relative to the wild-type (wt) constants**

| $k_{-1}^{1}=\alpha_{1}k_{-1}$ | $k_{2}^{1}=\beta_{1}k_{2}$ | $k_{1}^{1}=\gamma_{1}k_{1}$ |
| --- | --- | --- |
| $k_{-1}^{2}=\alpha_{2}k_{-1}$ | $k_{2}^{2}=\beta_{2}k_{2}$ | $k_{1}^{2}=\gamma_{2}k_{1}$ |

* Where $\alpha_{i},\beta_{i}, \gamma_{i}>0$

Thus the $K_{M}$ for each single mutant is:

|  | $K_{M}^{i}=\frac{\alpha_{i}k_{-1}+\beta_{i}k_{2}}{\gamma_{i}k_{1}}$ | (3) |
| --- | --- | --- |

And the fold-change (*f*) in *K*_M_ of a single mutant is:

|  | $f_{i}\equiv\frac{K_{M}^{i}}{K_{M}}=\frac{\alpha_{i}k_{-1}+\beta_{i}k_{2}}{\gamma_{i}{(k}_{1}+k_{2})}$ | (4) |
| --- | --- | --- |

Computing the observed fold-change of the double mutant can be made using the microscopic rate constant changes for the double mutant:

|  | $f_{1,2}^{obs}\equiv\frac{K_{M}^{1,2}}{K_{M}}=\frac{\alpha_{1}\alpha_{2}k_{-1}+\beta_{1}\beta_{2}k_{2}}{\gamma_{1}\gamma_{2}{(k}_{1}+k_{2})}$ | (5) |
| --- | --- | --- |

Whereas for the null model, we assume the fold-change of the double mutant is the product of the fold changes of each

|  | $f_{1,2}^{null}\equiv f_{1}f_{2}=\frac{{(\alpha}_{1}k_{-1}+\beta_{1}k_{2}){(\alpha}_{2}k_{-1}+\beta_{2}k_{2})}{\gamma_{1}\gamma_{2}{{(k}_{1}+k_{2})}^{2}}$ | (6) |
| --- | --- | --- |

***1.2 Necessary conditions for the presence of epistasis in K_M_***

The two fold-changes computed using observed rate constant differences versus the null model are only equal when:

|  | $\frac{\alpha_{1}\alpha_{2}k_{-1}+\beta_{1}\beta_{2}k_{2}}{\gamma_{1}\gamma_{2}{(k}_{1}+k_{2})}=\frac{{(\alpha}_{1}k_{-1}+\beta_{1}k_{2}){(\alpha}_{2}k_{-1}+\beta_{2}k_{2})}{\gamma_{1}\gamma_{2}{{(k}_{1}+k_{2})}^{2}}$ | (7) |
| --- | --- | --- |
|  | ${(\alpha}_{1}\alpha_{2}k_{-1}+\beta_{1}\beta_{2}k_{2}){(k}_{1}+k_{2})={(\alpha}_{1}k_{-1}+\beta_{1}k_{2}){(\alpha}_{2}k_{-1}+\beta_{2}k_{2})$ | (8) |
|  | ${(\alpha}_{1}\alpha_{2}+\beta_{1}\beta_{2})={(\alpha}_{1}\beta_{2}+\alpha_{2}\beta_{1})$ | (9) |
|  | ${(\alpha}_{1}-\beta_{1}){(\alpha}_{2}-\beta_{2})=0$ | (10) |

Thus, the null model fails and is distorted by epistasis when the fold-change in $k_{2}$ (*α*) or $k_{-1}$ (*β*) for at least one of the mutants is not equivalent, *i.e.,* $\alpha_{1}=\beta_{1}$ or $\alpha_{2}=\beta_{2}$

The same is logic can be applied to catalytic efficiency for the simple model, as it includes the summation expression $k_{-1}+k_{2}$from *K*_M_:

|  | $\frac{k_{cat}}{K_{M}}=\frac{k_{1}k_{2}}{k_{-1}+k_{2}}$ | (11) |
| --- | --- | --- |

***1.3 The effect of α and β on non-specific epistasis in K_M_***

Epistasis in *K*_M_ can be defined as:

|  | $\varepsilon_{K_{M}}=\frac{f_{1,2}^{obs}}{f_{1,2}^{null}}$ | (12) | |
| --- | --- | --- | --- |
|  | $\varepsilon_{K_{M}}= \frac{\frac{\alpha_{1}\alpha_{2}k_{-1}+\beta_{1}\beta_{2}k_{2}}{\gamma_{1}\gamma_{2}{(k}_{1}+k_{2})}}{\frac{{(\alpha}_{1}k_{-1}+\beta_{1}k_{2}){(\alpha}_{2}k_{-1}+\beta_{2}k_{2})}{\gamma_{1}\gamma_{2}{{(k}_{1}+k_{2})}^{2}}}$ | | (13) |

Which can be simplified to

|  | $\varepsilon_{K_{M}}= \frac{(\alpha_{1}\alpha_{2}k_{-1}+\beta_{1}\beta_{2}k_{2})}{\frac{{(\alpha}_{1}k_{-1}+\beta_{1}k_{2}){(\alpha}_{2}k_{-1}+\beta_{2}k_{2})}{k_{1}+k_{2}}}$ | (14) |
| --- | --- | --- |

Where $\varepsilon_{K_{M}}=1$ indicates no epistasis and $\varepsilon_{K_{M}}>1$ is positive epistasis and $\varepsilon_{K_{M}}<1$ is negative epistasis.

**2. Justification for the lack of epistasis in *k_cat_* for the simple model**

The single and double mutant *k_cat_* can be defined as follows:

|  | $k_{cat}^{1}=\beta_{1}k_{2}$  $k_{cat}^{2}=\beta_{2}k_{2}$  $k_{cat}^{1,2}={\beta_{1}\beta}_{2}k_{2}$ | (15) |
| --- | --- | --- |

And the fold-changes for the single mutant, as well as the predicted versus observed fold-changes for the double mutant:

|  | $f_{i}=\frac{k_{cat}^{i}}{k_{cat}^{wt}}=\frac{\beta_{i}k_{2}}{k_{2}}=\beta_{i}$ | (16) |
| --- | --- | --- |
|  | $f_{1,2}^{obs}= \frac{\beta_{1}\beta_{2}k_{2}}{k_{2}}=\beta_{1}\beta_{2}$ | (17) |
|  | $f_{1,2}^{null}=f_{1}f_{2}= \beta_{1}\beta_{2}=f_{1,2}^{obs}$ | (18) |

There is no apparent epistasis as the null model accurately captures the expected double-mutant fold-change.

**3. Justification for the lack of epistasis in *K*_D_ in all models**

Regardless of model complexity, the enzyme-substrate dissociation constant *K*_D_ is always defined as the equilibrium constant between the substrate-free and substrate bound enzyme complex:

|  | $K_{D}=\frac{\left[ E \right][S]}{[ES]}$ | (19) |
| --- | --- | --- |

Or, using microscopic rate constants:

|  | $K_{D}=\frac{k_{-1}}{k_{1}}$ | (20) |
| --- | --- | --- |

And the fold-changes for the single mutant, as well as the predicted versus observed fold-changes for the double mutant:

|  | $K_{D}^{i}=\frac{\alpha_{i}k_{-1}}{\gamma_{i}k_{1}}$ | (21) |
| --- | --- | --- |
|  | $f_{i}=\frac{K_{D}^{i}}{K_{D}}=\frac{\alpha_{i}k_{-1}k_{1}}{\gamma_{i}k_{1}k_{-1}}=\frac{\alpha_{i}}{\gamma_{i}}$ | (22) |
|  | $f_{1,2}^{obs}=\frac{K_{D}^{1,2}}{K_{D}}=\frac{\alpha_{1}\alpha_{2}}{\gamma_{1}\gamma_{2}}$ | (23) |
|  | $f_{1,2}^{null}=\frac{\alpha_{1}\alpha_{2}}{\gamma_{1}\gamma_{2}}=f_{1,2}^{obs}$ | (24) |

As with *k_cat_*_,_ we see no apparent epistasis. The lack of a summation term ensures the product of the fold-change in single mutational effects is equivalent to the observed fold-change. This will always result in no apparent epistasis.
