# Supplementary material for: Free energy perturbations in enzyme kinetic models reveal cryptic epistasis
Source: PLoS Comput Biol. 2026 Mar 11;22(3):e1013493. doi: 10.1371/journal.pcbi.1013493 (PMC12998945; doi:10.1371/journal.pcbi.1013493)
Supplement: S3 File — The Rmarkdown notebook outlining all of the code, with annotations, used to set up the more complex kinetic model (outlined in the Methods and Fig 4). The document outlines the results and conclusions drawn from the simulation. (HTML) [file pcbi.1013493.s003.html]

Complex kinetic model simulations


Code 

- Show All Code
- Hide All Code

# Complex kinetic model simulations

# 1 Overview

We first set up a free energy diagram for the the more complex
reaction E + S = ES = EP -> E + P. Then I extract rate and
equilibrium constants using the free energies, and make the assumption
for rate constants that the Arrhenius constant is *k*b
\* T / *h*.

Next, we introduce 1000 random “mutations” which sample from -2 to 2
kcal mol-1 and modulate the energy of each ground and/or
transition state by that amount. These are all save in a spreadsheet
(see Supplementary data 1).

Finally, we test all combinations of the 1000 mutations and calculate
the predicted and observed rate constants.

# 2 Setup

First we need to setup the free energy profile based on the simple
reaction E + S = ES = EP -> E + P

The free energies will be set up in the energies vector

```
energies <- list(e = 0,
                 e_s_dagger = 10,
                 es = -5,
                 es_dagger = 11,
                 ep = -9,
                 ep_dagger = 9
)
```

Then we define the global constants

```
kb = 3.297623483e-27 # kcal/K
h = 1.58e-37 # kcal s
temp = 273+25 # K in room temp
r = 1.9872036e-3 # kcal K-1 mol-1
A = (kb*temp)/h # Collision constant as defined broadly by transition state theory
```

We note that a more accurate approximation of the *A* constant
would utilize collision theory, due to the nature of E + S representing
a bimolecular collision of E + S. This means that the rate constant can
theoretically approach 6.2195683^{12} which exceeds rate-limiting
diffusion. Nevertheless, we retain this approximation for
simplicity.

Then we can define the equations for rate constants using the energy
terms:

```
define_constants <- function(energies) {
  kon = A*exp(-1*(energies$e_s_dagger-energies$e) / (r*temp)) # M s-1
  koff = A*exp(-1*(energies$e_s_dagger-energies$es) / (r*temp)) # s-1
  kchem = A*exp(-1*(energies$es_dagger-energies$es) / (r*temp)) # s-1
  kchemrev = A*exp(-1*(energies$es_dagger-energies$ep) / (r*temp)) # s-1
  kprod = A*exp(-1*(energies$ep_dagger-energies$ep) / (r*temp)) # s-1
  
  kd = koff/kon
  kcat = (kchem*kprod)/(kchem + kchemrev + kprod)
  km = ((kchem*kprod) + (koff*kchemrev) + (koff*kprod))/(kon*(kchem + kchemrev + kprod))
  catef = kcat/km
  
  return(list(kon = kon, 
              koff = koff, 
              kchem = kchem,
              kchemrev = kchemrev,
              kprod = kprod,
              kd = kd, 
              kcat = kcat, 
              km = km,
              catef = catef))
}
```

And check the values for each constants

```
cur_const <- define_constants(energies)
cur_const
```

```
## $kon
## [1] 288414.7
## 
## $koff
## [1] 62.10776
## 
## $kchem
## [1] 11.4755
## 
## $kchemrev
## [1] 0.0133744
## 
## $kprod
## [1] 0.3917626
## 
## $kd
## [1] 0.0002153419
## 
## $kcat
## [1] 0.3784033
## 
## $km
## [1] 8.655303e-06
## 
## $catef
## [1] 43719.24
```

These seem like appropriate starting conditions for the experiment
with *k*on ~ 2.8e5 M s-1,
*k*off ~ 60 s-1, *k*cat ~
0.378 s-1, *K*M ~ 8.6 µM,
*K*D ~ 215 µM, and a *k*cat /
*K*M of 4.3e4 M-1 s-1. The
*k*cat is low, while *k*cat /
*K*M lie within ranges of median enzymes.

# 3 Mutational simulations

First we prepare a table to store the free energies and rate
constants of the mutants:

```
dt <- tibble(e = numeric(),
             e_s_dagger = numeric(),
             es = numeric(),
             es_dagger = numeric(),
             ep = numeric(),
             ep_dagger = numeric(),
             kon = numeric(),
             koff = numeric(),
             kchem = numeric(),
             kchemrev = numeric(),
             kprod = numeric(),
             kd = numeric(),
             kcat = numeric(),
             km = numeric(),
             catef = numeric())

dt <- rbind(dt, c(unlist(energies), unlist(cur_const)))
names(dt) = c("e",
              "e_s_dagger",
              "es",
              "es_dagger",
              "ep",
              "ep_dagger",
              "kon",
              "koff",
              "kchem",
              "kchemrev",
              "kprod",
              "kd",
              "kcat",
              "km",
              "catef")
dt
```

```
##   e e_s_dagger es es_dagger ep ep_dagger      kon     koff   kchem  kchemrev
## 1 0         10 -5        11 -9         9 288414.7 62.10776 11.4755 0.0133744
##       kprod           kd      kcat           km    catef
## 1 0.3917626 0.0002153419 0.3784033 8.655303e-06 43719.24
```

Then we can setup a simulation where each mutant free energy is the
sum of the wt free energy and a randomly chosen energy ranging from -2
to 2 kcal mol-1

```
mutation <- function(dt){
  # Assuming mutation can change all steps by a random amount
  new_energies <- list(e = energies$e + runif(1, -2, 2),
                   e_s_dagger = energies$e_s_dagger + runif(1, -2, 2),
                   es = energies$es + runif(1, -2, 2),
                   es_dagger = energies$es_dagger + runif(1, -2, 2),
                   ep = energies$ep + runif(1, -2, 2),
                   ep_dagger = energies$ep_dagger + runif(1, -2, 2))
  mut_const <- define_constants(new_energies)
  
  return(rbind(dt, c(unlist(new_energies), unlist(mut_const))))
}
```

And we simulate 1000 mutations and export the data into
**Supplementary data 3**

```
for(i in 1:1000){
  dt <- mutation(dt)
}

head(dt)
```

```
##            e e_s_dagger        es es_dagger         ep ep_dagger         kon
## 1  0.0000000  10.000000 -5.000000 11.000000  -9.000000  9.000000   288414.66
## 2  1.8235750  11.749142 -6.047118 10.020295  -9.437952  8.364719   327042.77
## 3 -0.1904776   9.159731 -5.197307 12.226383  -8.577116  8.451364   864086.89
## 4  1.0653539   8.180348 -4.631312  9.806529  -8.469048  8.614360 37653611.58
## 5 -0.8379864  10.561836 -4.456129 12.959514  -7.275304  8.942019    27128.36
## 6  0.2987570  11.004953 -3.029110 10.723458 -10.502540  9.377041    87520.81
##           koff      kchem    kchemrev      kprod           kd       kcat
## 1   62.1077597  11.475499 0.013374403 0.39176261 2.153419e-04 0.37840327
## 2    0.5526376  10.240775 0.033387382 0.54668680 1.689802e-06 0.51738051
## 3  183.9405066   1.036750 0.003443572 2.02074134 2.128727e-04 0.68443247
## 4 2500.4487171 160.479158 0.245992665 1.84173901 6.640661e-05 1.81808686
## 5   60.2519047   1.050934 0.008996324 7.95046423 2.220993e-03 0.92730843
## 6  317.3502671 510.481960 0.001687172 0.01639029 3.625998e-03 0.01638971
##             km        catef
## 1 8.655303e-06   43719.2399
## 2 1.672582e-06  309330.4547
## 3 1.415640e-04    4834.7909
## 4 9.010977e-07 2017635.6764
## 5 1.996128e-03     464.5535
## 6 3.156676e-07   51920.7757
```

```
write_csv(dt, "Supplementary Data 3.csv")
```

# 4 General statistics

We interrogate the spread of *K*D,
*k*cat, *K*M, and
*k*cat / *K*M for the 1000 single
mutants (see **Fig. 4b** in the main text)

```
dt_all_spread <- dt %>%
  mutate(kcat_norm = log10(kcat / kcat[1]),
         kd_norm = log10(kd / kd[1]),
         km_norm = log10(km / km[1]),
         catef_norm = log10(catef / catef[1])) %>%
  pivot_longer(c(kcat_norm, kd_norm, km_norm, catef_norm)) %>%
  select(name, value)

# Change density plot fill colors by groups
dt_all_spread_plot <- dt_all_spread %>%
  ggplot(aes(x = value, fill = name)) +
  geom_density() +
  scale_fill_manual(values = c("#745fe8","#ca3a7d","#ea692f","#f3b33e")) +
  geom_vline(xintercept = log10(1), col = "black", lty = 2) +
  xlim(c(-5, 5)) +
  labs(x = "log10 parameter value", y = "Density") +
  theme_classic()
  #theme(axis.line = element_line(size = 0.2, color = "black"), axis.ticks = element_line(size = 0.2, color = "black"), text = element_text(size = 9), axis.text = element_text(size = 8, color = "black"), legend.position = "none")

dt_all_spread_plot
```

```
#ggsave("density_plot_complex.svg", plot = dt_all_spread_plot, width = 180/2, height = 247/4, dpi = 300, units = "mm")
```

# 5 Simulation of mutational combinations

Next, we create 106 double mutants by combining mutational
effects from each single mutant found in **Supplementary data
3**. We compute the predicted kinetic parameters and observed
parameters as follows:

For predicted kinetic parameters, we take the product of the wt
kinetic parameter, the fold-change of mutation 1, and the fold-change of
mutation 2. For the observed kinetic parameter, we first compute each
microscopic rate constant of the double mutant by calculating the sum of
free energy changes to each state in the reaction coordinate based on
the single mutation effects. Then, we use the newly computed rate
constants to obtained the *true* kinetic parameter of the double
mutant.

We quantify epistasis as the ratio of the predicted vs observed
kinetic parameter, and consider it significant if it exceeds
1.5-fold.

For each kinetic parameter, we also collect information on the fold
change of the single mutation effect to determine whether the double
mutant exhibits sign or magnitude epistasis.

```
library(purrr)
library(progress)

# Check for file, you can just upload if this is the case
if(file.exists('Supplementary Data 4.csv')){
  epi_dt <- read_csv("Supplementary Data 4.csv")
} else {
  # Generate all pairwise combinations of mutations
mut_combinations <- combn(2:(nrow(dt) - 1), 2, simplify = FALSE)

# Initialize a progress bar - ONLY WORKS IN CONSOLE, NOT IN RMD
pb <- progress_bar$new(
  format = "  Processing [:bar] :percent in :elapsed, ETA: :eta",
  total = length(mut_combinations),
  clear = FALSE,
  width = 60
)

# Process combinations with progress bar (console only)
epi_dt <- map_dfr(mut_combinations, function(pair) {
  pb$tick()  # Update progress bar at each iteration
  i <- pair[1]
  j <- pair[2]
  
  wt_catef <- dt$catef[1]
  mut1_fold_catef <- dt$catef[i] / wt_catef
  mut2_fold_catef <- dt$catef[j] / wt_catef
  
  pred_catef <- wt_catef * mut1_fold_catef * mut2_fold_catef
  
  wt_km <- dt$km[1]
  mut1_fold_km <- dt$km[i] / wt_km
  mut2_fold_km <- dt$km[j] / wt_km
  
  pred_km <- wt_km * mut1_fold_km * mut2_fold_km
  
  wt_kd <- dt$kd[1]
  mut1_fold_kd <- dt$kd[i] / wt_kd
  mut2_fold_kd <- dt$kd[j] / wt_kd
  
  pred_kd <- wt_kd * mut1_fold_kd * mut2_fold_kd
  
  wt_kcat <- dt$kcat[1]
  mut1_fold_kcat <- dt$kcat[i] / wt_kcat
  mut2_fold_kcat <- dt$kcat[j] / wt_kcat
  
  pred_kcat <- wt_kcat * mut1_fold_kcat * mut2_fold_kcat
  
  mut_e <- dt$e[1] + (dt$e[i] - dt$e[1] + dt$e[j] - dt$e[1])
  mut_e_s_dagger <- dt$e_s_dagger[1] + (dt$e_s_dagger[i] - dt$e_s_dagger[1] + dt$e_s_dagger[j] - dt$e_s_dagger[1])
  mut_es <- dt$es[1] + (dt$es[i] - dt$es[1] + dt$es[j] - dt$es[1])
  mut_es_dagger <- dt$es_dagger[1] + (dt$es_dagger[i] - dt$es_dagger[1] + dt$es_dagger[j] - dt$es_dagger[1])
  mut_ep <- dt$ep[1] + (dt$ep[i] - dt$ep[1] + dt$ep[j] - dt$ep[1])
  mut_ep_dagger <- dt$ep_dagger[1] + (dt$ep_dagger[i] - dt$ep_dagger[1] + dt$ep_dagger[j] - dt$ep_dagger[1])
  
  mut_energies <- list(e = mut_e, e_s_dagger = mut_e_s_dagger, es = mut_es, es_dagger = mut_es_dagger, ep = mut_ep, ep_dagger = mut_ep_dagger)
  cur_mut_const <- define_constants(mut_energies)
  
  tibble(
    e = mut_energies$e,
    e_s_dagger = mut_energies$e_s_dagger,
    es = mut_energies$es,
    es_dagger = mut_energies$es_dagger,
    ep = mut_energies$ep,
    ep_dagger = mut_energies$ep_dagger,
    kon = cur_mut_const$kon,
    koff = cur_mut_const$koff,
    kchem = cur_mut_const$kchem,
    kchemrev = cur_mut_const$kchemrev,
    kprod = cur_mut_const$kprod,
    kd = cur_mut_const$kd,
    kcat = cur_mut_const$kcat,
    km = cur_mut_const$km,
    catef = cur_mut_const$catef,
    pred_catef = pred_catef,
    pred_km = pred_km,
    pred_kd = pred_kd,
    pred_kcat = pred_kcat,
    mut1_fold = mut1_fold_catef,
    mut2_fold = mut2_fold_catef,
    mut1_fold_kcat = mut1_fold_kcat,
    mut2_fold_kcat = mut2_fold_kcat,
    mut1_fold_km = mut1_fold_km,
    mut2_fold_km = mut2_fold_km,
    mut1_fold_kd = mut1_fold_kd,
    mut2_fold_kd = mut2_fold_kd,
    mut1 = i,
    mut2 = j
  )
})

head(epi_dt)

write_csv(epi_dt, "Supplementary Data 4.csv")
}
```

# 6 Investigation of mutational combinations

## 6.1 Significant epistasis in *k*cat / *K*M

First I establish a function to calculate significant epistasis and
classify it as magnitude, sign, and reciprocal sign

```
sig_epi_classification <- function(sig_thresh, epi_dt) {
  this_df <- epi_dt %>%
  mutate(sign_change = log10(catef/dt$catef[1]),
         sign_mut1 = log10(mut1_fold),
         sign_mut2 = log10(mut2_fold),
         epi = log10(catef/pred_catef)) %>%
  mutate(sign = case_when( ((epi > log10(sig_thresh) | epi < log10(1/sig_thresh)) & sign_mut1 > 0 & sign_mut2 > 0 & sign_change < 0) ~ "reciprocal",
                           ((epi > log10(sig_thresh) | epi < log10(1/sig_thresh)) & sign_mut1 < 0 & sign_mut2 < 0 & sign_change > 0) ~ "reciprocal",
                           ((epi > log10(sig_thresh) | epi < log10(1/sig_thresh)) & sign_mut1 > 0 & sign_mut2 < 0 & (sign_change > sign_mut1 | sign_change < sign_mut2) ) ~ "sign",
                           ((epi > log10(sig_thresh) | epi < log10(1/sig_thresh)) & sign_mut1 < 0 & sign_mut2 > 0 & (sign_change < sign_mut1 | sign_change > sign_mut2) ) ~ "sign",
                           (epi > log10(sig_thresh) | epi < log10(1/sig_thresh)) ~ "magnitude",
                           TRUE ~ "no epistasis"))
  
  return(this_df)
}
```

Then, we check much significant epistasis is there in
*k*cat / *K*M with a 1.5-fold
threshold?

```
epi_dt_2_sig <- sig_epi_classification(1.5, epi_dt)
epi_dt_2_sig %>% count(sign) %>% mutate(n / sum(n) * 100)
```

```
## # A tibble: 4 × 3
##   sign              n `n/sum(n) * 100`
##   <chr>         <int>            <dbl>
## 1 magnitude    196477           39.4  
## 2 no epistasis 263525           52.9  
## 3 reciprocal     4516            0.906
## 4 sign          33983            6.82
```

What about a 2-fold threshold?

```
epi_dt_2_sig <- sig_epi_classification(2, epi_dt)
epi_dt_2_sig %>% count(sign) %>% mutate(n / sum(n) * 100)
```

```
## # A tibble: 4 × 3
##   sign              n `n/sum(n) * 100`
##   <chr>         <int>            <dbl>
## 1 magnitude    138629           27.8  
## 2 no epistasis 326234           65.4  
## 3 reciprocal     4292            0.861
## 4 sign          29346            5.89
```

A 5-fold threshold?

```
epi_dt_2_sig <- sig_epi_classification(5, epi_dt)
epi_dt_2_sig %>% count(sign) %>% mutate(n / sum(n) * 100)
```

```
## # A tibble: 4 × 3
##   sign              n `n/sum(n) * 100`
##   <chr>         <int>            <dbl>
## 1 magnitude     31010            6.22 
## 2 no epistasis 454135           91.1  
## 3 reciprocal     2627            0.527
## 4 sign          10729            2.15
```

A 10-fold threshold?

```
epi_dt_2_sig <- sig_epi_classification(10, epi_dt)
epi_dt_2_sig %>% count(sign) %>% mutate(n / sum(n) * 100)
```

```
## # A tibble: 4 × 3
##   sign              n `n/sum(n) * 100`
##   <chr>         <int>            <dbl>
## 1 magnitude      8428            1.69 
## 2 no epistasis 485430           97.4  
## 3 reciprocal     1153            0.231
## 4 sign           3490            0.700
```

Note that sign epistasis does not disappear even at high significance
thresholds

## 6.2 Significant epistasis in *k*cat

Again, I setup a function to calculate significant epistasis and
classify it as magnitude, sign, and reciprocal sign, this time for
*k*cat

```
sig_epi_classification_kcat <- function(sig_thresh, epi_dt) {
  this_df <- epi_dt %>%
  mutate(sign_change = log10(kcat/dt$kcat[1]),
         sign_mut1 = log10(mut1_fold_kcat),
         sign_mut2 = log10(mut2_fold_kcat),
         epi = log10(kcat/pred_kcat)) %>%
  mutate(sign = case_when( ((epi > log10(sig_thresh) | epi < log10(1/sig_thresh)) & sign_mut1 > 0 & sign_mut2 > 0 & sign_change < 0) ~ "reciprocal",
                           ((epi > log10(sig_thresh) | epi < log10(1/sig_thresh)) & sign_mut1 < 0 & sign_mut2 < 0 & sign_change > 0) ~ "reciprocal",
                           ((epi > log10(sig_thresh) | epi < log10(1/sig_thresh)) & sign_mut1 > 0 & sign_mut2 < 0 & (sign_change > sign_mut1 | sign_change < sign_mut2) ) ~ "sign",
                           ((epi > log10(sig_thresh) | epi < log10(1/sig_thresh)) & sign_mut1 < 0 & sign_mut2 > 0 & (sign_change < sign_mut1 | sign_change > sign_mut2) ) ~ "sign",
                           (epi > log10(sig_thresh) | epi < log10(1/sig_thresh)) ~ "magnitude",
                           TRUE ~ "no epistasis"))
  
  return(this_df)
}
```

Then, we check much significant epistasis is there in
*k*cat / *K*M with a 1.5-fold
threshold?

```
epi_dt_2_sig <- sig_epi_classification_kcat(1.5, epi_dt)
epi_dt_2_sig %>% count(sign) %>% mutate(n / sum(n) * 100)
```

```
## # A tibble: 4 × 3
##   sign              n `n/sum(n) * 100`
##   <chr>         <int>            <dbl>
## 1 magnitude    141532            28.4 
## 2 no epistasis 312451            62.7 
## 3 reciprocal     9759             1.96
## 4 sign          34759             6.97
```

What about a 2-fold threshold?

```
epi_dt_2_sig <- sig_epi_classification_kcat(2, epi_dt)
epi_dt_2_sig %>% count(sign) %>% mutate(n / sum(n) * 100)
```

```
## # A tibble: 4 × 3
##   sign              n `n/sum(n) * 100`
##   <chr>         <int>            <dbl>
## 1 magnitude    102228            20.5 
## 2 no epistasis 355171            71.2 
## 3 reciprocal     9559             1.92
## 4 sign          31543             6.33
```

A 5-fold threshold?

```
epi_dt_2_sig <- sig_epi_classification_kcat(5, epi_dt)
epi_dt_2_sig %>% count(sign) %>% mutate(n / sum(n) * 100)
```

```
## # A tibble: 4 × 3
##   sign              n `n/sum(n) * 100`
##   <chr>         <int>            <dbl>
## 1 magnitude     41817             8.39
## 2 no epistasis 428030            85.9 
## 3 reciprocal     7885             1.58
## 4 sign          20769             4.17
```

A 10-fold threshold?

```
epi_dt_2_sig <- sig_epi_classification_kcat(10, epi_dt)
epi_dt_2_sig %>% count(sign) %>% mutate(n / sum(n) * 100)
```

```
## # A tibble: 4 × 3
##   sign              n `n/sum(n) * 100`
##   <chr>         <int>            <dbl>
## 1 magnitude     19681             3.95
## 2 no epistasis 459951            92.3 
## 3 reciprocal     5878             1.18
## 4 sign          12991             2.61
```

Note that sign epistasis does not disappear even at high significance
thresholds

## 6.3 Significant epistasis in *K*M

A setup of the function to calculate and classify epistasis for
*K*M

```
sig_epi_classification_km <- function(sig_thresh, epi_dt) {
  this_df <- epi_dt %>%
  mutate(sign_change = log10(km/dt$km[1]),
         sign_mut1 = log10(mut1_fold_km),
         sign_mut2 = log10(mut2_fold_km),
         epi = log10(km/pred_km)) %>%
  mutate(sign = case_when( ((epi > log10(sig_thresh) | epi < log10(1/sig_thresh)) & sign_mut1 > 0 & sign_mut2 > 0 & sign_change < 0) ~ "reciprocal",
                           ((epi > log10(sig_thresh) | epi < log10(1/sig_thresh)) & sign_mut1 < 0 & sign_mut2 < 0 & sign_change > 0) ~ "reciprocal",
                           ((epi > log10(sig_thresh) | epi < log10(1/sig_thresh)) & sign_mut1 > 0 & sign_mut2 < 0 & (sign_change > sign_mut1 | sign_change < sign_mut2) ) ~ "sign",
                           ((epi > log10(sig_thresh) | epi < log10(1/sig_thresh)) & sign_mut1 < 0 & sign_mut2 > 0 & (sign_change < sign_mut1 | sign_change > sign_mut2) ) ~ "sign",
                           (epi > log10(sig_thresh) | epi < log10(1/sig_thresh)) ~ "magnitude",
                           TRUE ~ "no epistasis"))
  
  return(this_df)
}
```

```
epi_dt_2_sig <- sig_epi_classification_km(1.5, epi_dt)
epi_dt_2_sig %>% count(sign) %>% mutate(n / sum(n) * 100)
```

```
## # A tibble: 4 × 3
##   sign              n `n/sum(n) * 100`
##   <chr>         <int>            <dbl>
## 1 magnitude    281998            56.6 
## 2 no epistasis 166935            33.5 
## 3 reciprocal     6892             1.38
## 4 sign          42676             8.56
```

What about a 2-fold threshold?

```
epi_dt_2_sig <- sig_epi_classification_km(2, epi_dt)
epi_dt_2_sig %>% count(sign) %>% mutate(n / sum(n) * 100)
```

```
## # A tibble: 4 × 3
##   sign              n `n/sum(n) * 100`
##   <chr>         <int>            <dbl>
## 1 magnitude    214408            43.0 
## 2 no epistasis 238776            47.9 
## 3 reciprocal     6750             1.35
## 4 sign          38567             7.74
```

A 5-fold threshold?

```
epi_dt_2_sig <- sig_epi_classification_km(5, epi_dt)
epi_dt_2_sig %>% count(sign) %>% mutate(n / sum(n) * 100)
```

```
## # A tibble: 4 × 3
##   sign              n `n/sum(n) * 100`
##   <chr>         <int>            <dbl>
## 1 magnitude     78920            15.8 
## 2 no epistasis 393278            78.9 
## 3 reciprocal     5398             1.08
## 4 sign          20905             4.19
```

A 10-fold threshold?

```
epi_dt_2_sig <- sig_epi_classification_km(10, epi_dt)
epi_dt_2_sig %>% count(sign) %>% mutate(n / sum(n) * 100)
```

```
## # A tibble: 4 × 3
##   sign              n `n/sum(n) * 100`
##   <chr>         <int>            <dbl>
## 1 magnitude     33908            6.80 
## 2 no epistasis 449661           90.2  
## 3 reciprocal     4032            0.809
## 4 sign          10900            2.19
```

Note that sign epistasis does not disappear even at high significance
thresholds

## 6.4 Significant epistasis in *K*D

```
epi_dt %>%
  mutate(sign_change = log10(kd/dt$kd[1]),
         epi = log10(kd/pred_kd)) %>%
  mutate(sign = case_when( (epi > log10(1.5) | epi < log10(1/1.5)) ~ "epistasis",
                           TRUE ~ "no epistasis")
         
  ) %>%
  count(sign) %>% mutate(n / sum(n) * 100)
```

```
## # A tibble: 1 × 3
##   sign              n `n/sum(n) * 100`
##   <chr>         <int>            <dbl>
## 1 no epistasis 498501              100
```

## 6.5 Correlation plots of predicted vs observed effects

What is the plot of *k*cat / *K*M
of predicted vs observed employing a 1.5-fold threshold?

```
epi_dt_2_sig <- sig_epi_classification(1.5, epi_dt)

epi_dt_2_sig_catef_plot <- epi_dt_2_sig %>%
  mutate(sign = factor(sign, levels = c("no epistasis", "magnitude", "sign", "reciprocal"))) %>%
  ggplot(aes(x = log10(pred_catef), y = log10(catef), color = sign)) +
  geom_point(alpha = 0.4, size = 0.5) +
  geom_abline(slope = 1, intercept = 0, linewidth = 0.3) +
  geom_hline(yintercept = log10(dt[1,]$catef), lty = 2, linewidth = 0.3) +
  geom_vline(xintercept = log10(dt[1,]$catef), lty = 2, linewidth = 0.3) +
  scale_color_manual(values = c("grey","#E69F00","#1f78b4","#d73027")) +
  labs(x = "Log10(Predicted kcat/Km)", "Log10(Observed kcat/Km)") +
  theme_classic() +
  theme(axis.line = element_line(size = 0.3, color = "black"), axis.ticks = element_line(size = 0.2, color = "black"), text = element_text(size = 9), axis.text = element_text(size = 8, color = "black"))
```

```
## Warning: The `size` argument of `element_line()` is deprecated as of ggplot2 3.4.0.
## ℹ Please use the `linewidth` argument instead.
## This warning is displayed once every 8 hours.
## Call `lifecycle::last_lifecycle_warnings()` to see where this warning was
## generated.
```

```
epi_dt_2_sig_catef_plot
```

```
#ggsave("catef_muts_comp.tiff", plot = epi_dt_2_sig_catef_plot, width = 180/2, height = 247/4, dpi = 600, units = "mm")
```

What is the plot of *K*M predicted vs observed?

```
epi_dt_2_sig_km <- sig_epi_classification_km(1.5, epi_dt)

epi_dt_2_sig_km_plot <- epi_dt_2_sig_km %>%
  mutate(sign = factor(sign, levels = c("no epistasis", "magnitude", "sign", "reciprocal"))) %>%
  ggplot(aes(x = log10(pred_km), y = log10(km), color = sign)) +
  geom_point(alpha = 0.4, size = 0.5) +
  geom_abline(slope = 1, intercept = 0, linewidth = 0.3) +
  geom_hline(yintercept = log10(dt[1,]$km), lty = 2, linewidth = 0.3) +
  geom_vline(xintercept = log10(dt[1,]$km), lty = 2, linewidth = 0.3) +
  scale_color_manual(values = c("grey","#E69F00","#1f78b4","#d73027")) +
  labs(x = "Log10(Predicted Km)", "Log10(Observed Km)") +
  theme_classic() +
  theme(axis.line = element_line(size = 0.3, color = "black"), axis.ticks = element_line(size = 0.2, color = "black"), text = element_text(size = 9), axis.text = element_text(size = 8, color = "black"))

epi_dt_2_sig_km_plot
```

What is the plot of *k*cat predicted vs
observed?

```
epi_dt_2_sig_kcat <- sig_epi_classification_kcat(1.5, epi_dt)

epi_dt_2_sig_kcat_plot <- epi_dt_2_sig_kcat %>%
  mutate(sign = factor(sign, levels = c("no epistasis", "magnitude", "sign", "reciprocal"))) %>%
  ggplot(aes(x = log10(pred_kcat), y = log10(kcat), color = sign)) +
  geom_point(alpha = 0.4, size = 0.5) +
  geom_abline(slope = 1, intercept = 0, linewidth = 0.3) +
  geom_hline(yintercept = log10(dt[1,]$kcat), lty = 2, linewidth = 0.3) +
  geom_vline(xintercept = log10(dt[1,]$kcat), lty = 2, linewidth = 0.3) +
  scale_color_manual(values = c("grey","#E69F00","#1f78b4","#d73027")) +
  labs(x = "Log10(Predicted kcat)", "Log10(Observed kcat)") +
  theme_classic() +
  theme(axis.line = element_line(size = 0.3, color = "black"), axis.ticks = element_line(size = 0.2, color = "black"), text = element_text(size = 9), axis.text = element_text(size = 8, color = "black"))

epi_dt_2_sig_kcat_plot
```

```
#ggsave("kcat_muts_comp_model.tiff", plot = epi_dt_2_sig_kcat_plot, width = 180/2, height = 247/4, dpi = 600, units = "mm")
```

What is the plot of *K*D predicted vs observed?

```
epi_dt_2_sig_kd <- epi_dt %>%
  mutate(sign_change = log10(kd/dt$kd[1]),
         epi = log10(kd/pred_kd)) %>%
  mutate(sign = case_when( (epi > log10(1.5) | epi < log10(1/1.5)) ~ "epistasis",
                           TRUE ~ "no epistasis"))

# View(dt %>% mutate(across(everything(), ~ . - first(.))))

epi_dt_2_sig_kd_plot <- epi_dt_2_sig_kd %>%
  ggplot(aes(x = log10(pred_kd), y = log10(kd), color = sign)) +
  geom_point(alpha = 0.4) +
  geom_abline(slope = 1, intercept = 0, linewidth = 0.3) +
  geom_hline(yintercept = log10(dt[1,]$kd), lty = 2, linewidth = 0.3) +
  geom_vline(xintercept = log10(dt[1,]$kd), lty = 2, linewidth = 0.3) +
  scale_color_manual(values = c("grey")) +
  labs(x = "Log10(Predicted Kd)", "Log10(Observed Kd)") +
  theme_classic() +
  theme(axis.line = element_line(size = 0.3, color = "black"), axis.ticks = element_line(size = 0.2, color = "black"), text = element_text(size = 9), axis.text = element_text(size = 8, color = "black"))

epi_dt_2_sig_kd_plot
```

## 6.6 Positive-negative spread

What is the spread of positive and negative mutations for
*k*cat / *K*M

```
epi_dt %>%
  mutate(epi = log10(catef/pred_catef)) %>%
  filter(epi >= log10(1.5) | epi <= log10(1/1.5)) %>%
  mutate(posneg = case_when(epi > 0 ~ "positive",
                           epi < 0  ~ "negative",
                           TRUE ~ "neutral")) %>%
  count(posneg) %>%
  mutate(freq = n / sum(n) * 100)
```

```
## # A tibble: 2 × 3
##   posneg        n  freq
##   <chr>     <int> <dbl>
## 1 negative  91916  39.1
## 2 positive 143060  60.9
```

Then *K*M

```
epi_dt %>%
  mutate(epi = log10(km/pred_km)) %>%
  filter(epi >= log10(1.5) | epi <= log10(1/1.5)) %>%
  mutate(posneg = case_when(epi > 0 ~ "positive",
                           epi < 0  ~ "negative",
                           TRUE ~ "neutral")) %>%
  count(posneg) %>%
  mutate(freq = n / sum(n) * 100)
```

```
## # A tibble: 2 × 3
##   posneg        n  freq
##   <chr>     <int> <dbl>
## 1 negative 180524  54.4
## 2 positive 151042  45.6
```

Then *k*cat

```
epi_dt %>%
  mutate(epi = log10(kcat/pred_kcat)) %>%
  filter(epi >= log10(1.5) | epi <= log10(1/1.5)) %>%
  mutate(posneg = case_when(epi > 0 ~ "positive",
                           epi < 0  ~ "negative",
                           TRUE ~ "neutral")) %>%
  count(posneg) %>%
  mutate(freq = n / sum(n) * 100)
```

```
## # A tibble: 2 × 3
##   posneg       n  freq
##   <chr>    <int> <dbl>
## 1 negative 90208  48.5
## 2 positive 95842  51.5
```
