# Supplementary material for: Free energy perturbations in enzyme kinetic models reveal cryptic epistasis
Source: PLoS Comput Biol. 2026 Mar 11;22(3):e1013493. doi: 10.1371/journal.pcbi.1013493 (PMC12998945; doi:10.1371/journal.pcbi.1013493)
Supplement: Attachment — Submitted filename: Response to Reviewers.pdf [file pcbi.1013493.s005.pdf]

THE UNIVERSITY OF BRITISH COLUMBIA

The University of British Columbia
Nobuhiko Tokuriki, Ph.D.
Professor
Michael Smith Laboratories, UBC
327 - 2185 East Mall
Vancouver, BC, V6T 1Z4, Canada
Phone: +1 604 822 8156
Fax: +1 604 822 2114
Email: tokuriki@msl.ubc.ca

December 9th, 2025

Ref: Decision on Manuscript PCOMPBIOL-D-25-01794

Dear Dr. Eric C. Dykeman,

We thank you very much for communicating the reviewers' comments regarding our manuscript entitled "*Free energy perturbations in enzyme kinetic models reveal cryptic epistasis*". We are grateful to see the interest of the reviewers, and we would like to thank them for their constructive comments to further improve the manuscript.

You will find enclosed a point-by-point response to these comments. Each point is supported by line number references of the revised text as well as in-text edits in the "track changes" file marked in red (note, we omitted tracking re-numbering of references unless we included new references or modified the text that cites previous references). Additionally, we have provided a "clean" version of the manuscript, as requested, ready for publication, should our revised article be accepted.

We would be extremely grateful for a prompt decision on our submission, ideally by the end of this calendar year. The urgency stems from the fact that the University of British Columbia's contract with PLOS is expiring. If a decision is not finalized before December 31, 2025, I will lose access to the institutional fee waiver and incur the cost of the Article Processing Charge myself.

We hope that you will find our revised manuscript suitable for publication. Thank you again for your consideration.

Sincerely yours,

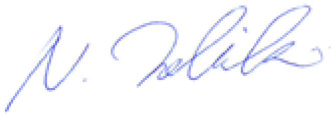

Nobuhiko Tokuriki, Ph.D.

Response to Reviewer #1

1.1 *This assumption [free energies that are assumed to change on an additive scale (that is, the free energy change associated with a double mutant is the sum of the free energy changes of the constituent single mutations)], which is central to the entire investigation, seems to me to be* ***completely unfounded****.*

We appreciate this comment from the reviewer, as our initial manuscript did not adequately support this assumption with references. This claim has long been held in the field, with the first in-depth exploration conducted by Wells in 1990 (PMID: 2271534). By analysing several multi-mutational protein datasets, including free energies of enzyme transition states, Wells found that "in the majority of cases, the sum of the free energy changes derived from the single mutations is nearly equal to the free energy change measured in the multiple mutant." Since then, Wells' study has been cited 1767 times, with the most recent citing study published in September 2025. Although recent investigations have received substantially less focus on the free energy additivity in enzymes compared to other proteins, there has been considerable effort in further demonstrating this assumption for protein folding and binding using contemporary, empirical methods (PMIDs: 25455030, 30037990, 39322666). These studies arrived at the same conclusion: in proteins, changes in free energies arising *via* mutations are mostly additive with a small contribution from pairwise energetic couplings (epistasis). Thus, we believe our central assumption is **founded on a comprehensive and robust body of empirical and theoretical work**. To strengthen the rationale for accepting this assumption, we have explicitly addressed the work supporting this assumption, with references provided, in the results (**lines 159-162**).

1.2 *The empirical example of the Bacillus cereus beta-lactamase that the authors discuss shows it (the assumption of additive free energy changes) to be* ***manifestly wrong****.*

Although we do not agree that this singular example demonstrates that the assumption outlined in response ***1.1*** is "manifestly wrong," we agree with the reviewer that the *B. cereus* β-lactamase example challenges the generality of this assumption, which is precisely the message we try to highlight in our work. The main source of epistasis in the *B. cereus* β-lactamase is the specific epistasis stemming from direct interactions, which greatly affects the microscopic rate constants within the double mutant (**lines 290-293**). This is the extensively explored source of epistasis outlined by Wells (PMID: 2271534): "…there are two major exceptions where such simple additivity breaks down. The first is where the mutated residues interact with each other, by direct contact or indirect contact through electrostatic interactions or structural perturbations…" However, our analysis enables the extraction of the weaker, yet still present, non-specific epistasis stemming from the non-linear mapping of microscopic rate constants to k_{cat} and K_M. Although in this specific case, the contribution of epistasis was weak, based on our simulations, these effects can reach several orders of magnitude (**Figs. 2–4**), hence the call-to-action for collecting more data to explore this form of non-specific epistasis (**lines 339-352**). Indeed, mutational interactions rooted in the enzyme structure are the predominant causal factor attributed to epistasis in k_{cat} and/or K_M in many studies (*e.g.*, PMIDs: 25902402, 30982891), even though, as we show, non-specific epistasis cannot and should not be excluded. In fact, Wells did raise the alternative scenario of deviation from additivity when "…the mutation causes a change in mechanism or rate-limiting step of the reaction," hinting at some form of non-specific epistasis. This latter point, brought up hypothetically by Wells, is a phenomenon we have observed experimentally in our previous work (PMID: 38828429). In this work, we expand on this observation and speculation by providing the exact conditions of how a shift in the rate-limiting step underpins the arising non-specific epistasis (outlined in **Figure 3** and **lines 187-224**). Thus, we believe that instead of disproving the free energy additivity assumption, **the *B. cereus* β-lactamase example outlines and dissects the factors that cause deviation from the free energy additivity assumption in enzymes**.

1.3 *The distinction between specific and non-specific epistasis is popular, but, to my mind,* ***not very helpful.***

For practitioners, one of the primary roles of distinguishing specific and non-specific epistasis is the ability to "correct for" the latter with appropriate models. The remaining variance is then attributed to specific positional effects, resulting in a model that accurately captures the architecture of the fitness landscape (PMID: 39261454). Thus, **we believe this distinction is helpful** as it allows for portioning of the contribution that global (phenotypic or biophysical) and idiosyncratic (position specific) factors play in navigating the fitness landscape; this is of great interest to researchers interested in prediction and/or engineering of new functions, or complementing measurements of specific epistasis with structural analyses to make mechanistic inferences on the role that the mutations play in the functional changes.

1.3 *But even if I accept the setting of the study as motivated by the [...] distinction between specific and non-specific epistasis, I feel that* ***the findings are of very limited interest****.*

Recent publications from the Harms lab have highlighted that an additional source of non-specific–ensemble epistasis–has the potential to create non-additive effects that cannot be easily captured by contemporary non-linear transformations, even though they are not caused by direct or indirect biophysical mutational interactions (PMID: 29078365, 34849909, 36710492). This series of peer-reviewed studies has been cited a total of 129 times, indicating substantial interest. Although foundational and elegant, their study necessitates the existence of a protein ensemble composed of at least three states, two of which must be inactive. This type of conformational ensemble is not the norm for most enzyme mechanisms, limiting the applicability of the model. However, by expanding on the theoretical overview of ensemble epistasis, we demonstrate that a similar form of **non-specific epistasis may, in principle, be found in all enzymes** by highlighting the fact that the catalytic cycle is, itself, an ensemble composed of three or more states (though not partitioned into active and inactive, as is the case for ensemble epistasis). This suggests that the existence of this form of **non-specific epistasis is always a possibility for enzyme kinetic parameters**. Therefore, our findings are relevant to any researcher analysing kinetic parameters of an enzyme with two or more mutations relative to a reference state, as well as any enzyme engineer attempting to predict the activity of an enzyme with two or more mutations. Thus, **our study should be very interesting and relevant to a broad audience**.

1.4 *What is announced* ***somewhat grandly as "in silico enzyme models"*** *amounts to evaluating the changes in simple rational functions, given explicitly in the Methods section, under random multiplicative changes of the constituent elementary transition rates.*

We appreciate that this phrasing may be misleading. Although technically correct, we see how it is possible that "*in silico* enzyme models" may be misconstrued to imply atomistic or lattice models, as opposed to the models we use in our study. Although described by the reviewer as "changes in simple rational functions," these functions are sufficient in defining a free energy model consisting of states within common enzyme mechanisms. By monitoring perturbations to these states *via* "random multiplicative changes of the constituent elementary transition rates," **these seemingly "simple" models are sufficient to draw novel and complex conclusions**, namely the discovery of a new source of non-specific epistasis in enzyme kinetic parameters. Nevertheless, to avoid confusion, we rephrased the term "*in silico* enzyme models" to "enzyme catalytic cycle models" in the abstract and discussion (**lines 16** and **313**)–the only two sections of the manuscript that used the old phrasing.

1.5 *Overall, the work* ***does not substantially advance our understanding of (specific or non-specific) epistasis****.*

We hope that all of the responses above convince the reviewer and editor otherwise. We also thank the reviewer for the constructive comments that have helped improve the clarity of the manuscript.

Response to Reviewer #2

2.1 *This is a very valuable contribution in the field of evolutionary genetics, dealing with how non-specific epistasis can arise from even relatively simple enzymatic reaction pathways because individual mutations can affect the energies of multiple reaction steps. Because these energies combine in non-linear ways to determine macroscopic reaction constants, this leads to apparent epistasis, even for mutations in which all effects on the level of energies are strictly additive. This paper builds on the idea of ensemble epistasis but is honest about that precedent. I find this work very relevant, because deep mutational scanning and quantification of epistasis is becoming an ever more widely used technique, especially in enzymes, and it seems very important to me that its practitioners are aware of the sources of epistasis in their datasets. [...] I judge this to be an important piece of work so did not want to pass up on the opportunity to review it.*

We appreciate the reviewer's concise and accurate summary of our manuscript, especially their last clause highlighting the timeliness of our findings. We would like to thank the reviewer for the time and energy invested in the feedback.

2.2 *In many enzymes the exact reaction pathway may not be known in all its complexity. The authors already show that taking care of the ensemble epistasis in one case can lead to different values for the specific epistasis in the system. How serious a problem is model mis-specification (i.e. a simplified kinetic scheme relative to the more complex biophysical reality) for our desire to quantify specific epistasis?*

We thank the reviewer for the astute observation and interesting question. Indeed, model mis-specification is very likely to be found in the literature for enzyme kinetic analyses, as many non-enzymologists may assume their enzyme follows the standard Michaelis-Menten model presented in **Figure 1**. In this case, if the researchers attempt to extract microscopic rate constants using various pre-steady state kinetic techniques to account for the non-specific epistasis we have outlined in our work, they may instead be measuring some k_{obs} or $k_{apparent}$–a composite of multiple underlying microscopic rate constants. These rates will be affected by non-specific epistasis in the same manner that macroscopic rate constants are, since mis-specified microscopic constants are, in principle, a type of macroscopic rate constant. We clarify and elaborate on this matter in **lines 338-342** of the updated manuscript.

2.3 *Another comment would be whether we have much evidence that individual mutations usually affect multiple energy levels along the reaction coordinate. It's not an unreasonable, assumption, but I am wondering whether the authors can cite more examples where this is known to be true.*

Indeed, supporting this assumption in our text would strengthen the rationale for our methodology–we thank the reviewer for this suggestion. This information can be inferred from the changes across multiple microscopic rate constants of single mutants, relative to the wild-type, across several enzymes such as DHFR (PMID: 7495797), OMPDC (PMID: 26135041), InhA (PMID: 16647717), DADH (PMID: 36795942), and TrpH (PMID: 29589922). Much like in our cited example of *B. cereus* β-lactamase, mutations that affect multiple free energy states seem to be the norm, not the exception. We have added an additional sentence in the methods to justify this assumption (**lines 149-151**), and provided all of the references listed above in the text.

Response to Reviewer #3

3.1 *In this work, the authors explore the properties of enzyme kinetic models and describe a property that they term cryptic epistasis, meaning that although mutations may appear to have linear effects on basic enzyme properties (free energies), they can combine in non-linear ways to affect observed kinetic properties such as Km and kcat. The paper is clearly written for the most part.*

We thank the reviewer for their apt summary of our work.

3.2 *Enzyme kinetic parameters (e.g. Km, kcat) are of course well-established to be non-linear combinations of microscopic reaction rate constants (e.g. k1, k2) or reaction free energies (dG or equivalently K), which are presumably what is affected by mutations affecting reaction step free energies. Thus, I am confused why it is surprising that affecting microscopic parameters would have a non-linear effect on macroscopic parameters. Can the authors better justify the current thinking in the field on this claim, with citations?*

As the reviewer states, this fact is, of course, not surprising. Nonetheless, contemporary analyses in the field currently map the changes in these macroscopic parameters directly to free energies (PMIDs: 25902402, 30982891… as well as the previously provided references in **line 328**). These parameters are then complemented by structural analyses to make direct inferences on specific epistasis, implying (albeit never explicitly stated) that these parameters are devoid of non-specific epistasis. Our findings, although based on the unsurprising fact that microscopic parameters map non-linearly to macroscopic parameters, demonstrate how strong and complex the arising non-linearity can be. **In summary, we have clarified the current thinking in the field on this claim in lines 112-114**. We also speculate on why the field has likely omitted considerations of non-specific epistasis in macroscopic parameters in **lines 319-328** and support our claim with citations listed above (**lines 328**).

3.3 *I am also unsure whether the authors can definitively state that a mutation would affect a single parameter (i.e. an individual free energy) in a linear/additive way with other mutations (if I understand their methodology correctly – feel free to correct me) – how realistic is this assumption itself? Can the authors assess this from the beta-lactamase case?*

We thank the reviewer for highlighting this. The rationale for the assumption is elaborated on in our response to reviewer #1 (see response ***1.1***) and addressed in **lines 159-163**. We do not find this assumption to hold true in our analysis of *B. cereus* β-lactamase; however, this is an exception where the non-linearity is caused by specific epistasis–what remains after the extraction of non-specific epistasis using our model (see response ***1.2*** and **lines 290-293** in our manuscript). We hope these responses and changes to the manuscript address the reviewer's concerns.

3.4 *Who says "Enzyme kinetic parameters are assumed to be devoid of non-specific epistasis when measured in vitro" – why would this be the case? What is the basis for this assumption? In the Discussion the authors state that it is commonly believed that "the confounding mutational effects on the physical properties of the enzyme can be controlled through appropriate experimental design," but I am having trouble even conceptualizing how experimental design of an enzyme assay could control the degree of epistasis of mutations within the enzyme on its observed macroscopic kinetic parameters (Km, kcat).*

This assumption appears to be implicit in the field, as epistasis in kinetic parameters is typically complemented by structural analyses to make direct inferences on specific epistasis, implying that these parameters are devoid of non-specific epistasis (PMIDs: 25902402, 30982891). We have touched on this in response ***3.2*** and **lines 112-114,** as well as **lines 319-328**. Nevertheless, as this assumption has never been explicitly stated in the references we provide, we have opted to alter the phrasing in the

abstract and the discussion in **lines 12-14** of the abstract and **lines 319-323** of the discussion (the latter also addresses revision request ***3.2***). We hope these changes clarify our intent to highlight the previous disregard of potential non-specific epistasis in enzyme kinetic parameters, as opposed to claiming that researchers have been explicitly assuming that non-specific epistasis is absent in these forms of data.

3.5 *In several places it seems to me the authors could better describe specific and non-specific epistasis. In particular, the authors state that non-specific epistasis is when a non-linear mapping is present, but isn't this the definition of epistasis in general, when two mutations combine in non-linear (i.e. non-additive) ways? Maybe a more precise phrasing would be possible here, to distinguish from epistasis where mutations physically interact, which is a clear concept of course.*

We thank the reviewer for pointing out the shortcomings of our current definitions. We have added an additional sentence to clarify the contrast between specific versus non-specific epistasis, namely non-linearity stemming from mutations that exhibit non-linear mapping despite additive/linear combination on the free energy scale (non-specific epistasis) *versus* non-linearity arising due to non-additive/non-linear changes in free energy upon combining mutations. We have made this change in **lines 80-83**.
