# Supplementary material for: Free energy perturbations in enzyme kinetic models reveal cryptic epistasis
Source: PLoS Comput Biol. 2026 Mar 11;22(3):e1013493. doi: 10.1371/journal.pcbi.1013493 (PMC12998945; doi:10.1371/journal.pcbi.1013493)
Supplement: Attachment — Submitted filename: rebuttal_letter_rev02_v02.pdf [file pcbi.1013493.s006.pdf]

THE UNIVERSITY OF BRITISH COLUMBIA

The University of British Columbia
Nobuhiko Tokuriki, Ph.D.
Professor
Michael Smith Laboratories, UBC
327 - 2185 East Mall
Vancouver, BC, V6T 1Z4, Canada
Phone: +1 604 822 8156
Fax: +1 604 822 2114
Email: tokuriki@msl.ubc.ca

February 9th, 2026

Ref: Decision on Manuscript PCOMPBIOL-D-25-01794R1

Dear Dr. Eric C. Dykeman,

We thank you very much for communicating the reviewers' comments regarding our manuscript entitled "*Free energy perturbations in enzyme kinetic models reveal cryptic epistasis*". We are grateful to see all the reviewers recommend our work for publication, and we would like to thank them for their contributions throughout the revision process.

You will find enclosed a response to the last two comments from reviewer #1. Each point is supported by line number references of the revised text as well as in-text edits in the "track changes" file marked in red (note, we omitted tracking re-numbering of references). Additionally, we have provided a "clean" version of the manuscript, as requested, ready for publication, should our revised article be accepted.

We hope that you will find our revised manuscript suitable for publication. Thank you again for your consideration.

Sincerely yours,

Nobuhiko Tokuriki, Ph.D.

<u>Response to Reviewer #1</u>

1.1 *The authors have provided detailed responses to all my comments. Although I remain unconvinced of the broader significance of the study, my specific points of criticism have been addressed, and I do not object to the publication of the manuscript in PloS Comp. Biol.*

We appreciate the in-depth feedback provided by the reviewer which greatly helped improve the clarity and flow of the manuscript.

1.2 *The empirical evidence for the additivity of transition state free energies (as presented in the new reference by Wells and other work) is a crucial basis for the study. Since it was unknown to me, it may be unknown to other readers as well. It should therefore be mentioned and discussed explicitly early in the introduction.*

We have added an explicit definition of this assumption with a reference to Wells' work in **lines 110-111** and retain the more in-depth, methodologically relevant explanation (with additional references) in **lines 161-165.**

1.3 *Similarly, the fact that the beta-lactamase system does not conform to the additive pattern (but does not disprove the latter, because it is known to be an exception) should be mentioned explicitly either in the Introduction or in the corresponding Results section, and it should be explained why the authors nevertheless choose to discuss this example.*

We have now explicitly mentioned why we expected the β-lactamase example to be an exception to the additivity rule (**lines 279-282**), as well as why, despite that, the study by Gibson & Waley was the best (and only appropriate) study that could be used to demonstrate the utility of our analysis (**lines 282-285**).

<u>Response to Reviewer #2</u>

2.1 *My concerns have been addressed.*

We would like to thank the reviewer for their help in improving the manuscript during the first round of revisions.

<u>Response to Reviewer #3</u>

3.1 *The authors have sufficiently clarified my areas of confusion in the previous draft. I have no additional concerns.*

We thank the reviewer for highlighting the weak points in our original manuscript during the first round of revisions.
